# Intermittent Two-Point Dynamics at the Transition to Chaos for Random Circle Endomorphisms

**DOI:** 10.1007/s00220-026-05565-w

**Published:** 2026-04-04

**Authors:** V. P. H. Goverse, A. J. Homburg, J. S. W. Lamb

**Affiliations:** 1 Department of Mathematics, Imperial College London, 180 Queen’s Gate, London, SW7 2AZ UK; 2 KdV Institute for Mathematics, University of Amsterdam, Science Park 107, 1098 XG Amsterdam, The Netherlands; 3 Mathematics Institute, Leiden University, Einsteinweg 55, 2333 CC Leiden, The Netherlands; 4 International Research Center for Neurointelligence (IRCN), The University of Tokyo, Tokyo, 113-0033 Japan; 5 Centre for Applied Mathematics and Bioinformatics, Department of Mathematics and Natural Sciences, Gulf University for Science and Technology, 32093 Halwally, Kuwait

## Abstract

We establish the existence of intermittent two-point dynamics and infinite stationary measures for a class of random circle endomorphisms with zero Lyapunov exponent, as a dynamical characterisation of the transition from synchronisation (negative Lyapunov exponent) to chaos (positive Lyapunov exponent).

## Introduction

In dynamical systems theory, the phenomenon of chaos (with hallmark sensitive dependence on initial conditions) has been an important motivation and focal point of research. In particular, the question of when and why chaotic dynamics emerges from more predictable motion, for instance in a parametrized family of dynamical systems, has been a central question in bifurcation theory.

While several routes to chaos have been identified for deterministic dynamical systems (see for instance [33]), the corresponding transition in random dynamical systems (dynamical systems driven by a signal with certain probabilistic characteristics) remains much less understood.

In this paper, we address this problem for a class of random circle endomorphisms, adopting notions of order and chaos in terms of the sign of the Lyapunov exponent, with negative Lyapunov exponent implying synchronisation (almost sure convergence of the distance between different trajectories with different initial conditions) and positive Lyapunov exponent implying chaos (including sensitive dependence on initial conditions). We are led to address the question how this transition happens, and in particular to identify dynamical aspects that accompany the change of sign of the Lyapunov exponent. We focus in this respect on the so-called two-point motion from the topological and (invariant) measure theoretical point of view.

In late 1980s, Baxendale and Stroock [11, 13] studied the dynamics of stochastic differential equations, characterising stationary measures for the two-point motion in different Lyapunov exponent regimes, establishing in particular the existence of an infinite ergodic invariant measure at the zero Lyapunov transition point between synchronisation (stationary probability measure on the diagonal) and chaos (stationary probability measures on and off the diagonal).

In the early 1990s, Pikovsky [60] and Yu, Ott and Chen [67] studied the transition to chaos in the discrete time setting (random maps), leading to numerical evidence supporting several heuristic conjectures concerning intermittency.

Recently, Homburg and Kalle [41] obtained explicit results about stationary measures for certain random affine iterated function systems on the circle. They also point out the fact that the infinite stationary measure of the two-point motion at the transition corresponds to intermittent dynamics.

This leads to the natural question whether intermittency and associated infinite ergodic stationary measure of the two-point motion are generic features of the transition to chaos in random dynamical systems.

In this paper, we answer this question in the affirmative, in the specific setting of circle endomorphisms of degree two with additive noise. Importantly, we develop techniques to analyse the two-point dynamics near the diagonal, expanding on results developed by Baxendale and Stroock [11, 13] for stochastic differential equations. In particular, we analyse the spectral properties and actions of annealed Koopman operators for one- and two-point motions, allowing us to derive quantitative estimates on various escape times of the quenched process, which are key to our results. In Sect. 2.2, we present a summary of our techniques.

We anticipate that the techniques introduced in this paper will turn out to be fundamental to settle our question in general.

### Main results

Consider a smooth monotone circle endomorphism $T: \mathbb {T} \rightarrow \mathbb {T}$ of degree $k>1$, where $\mathbb T = \mathbb {R}/\mathbb {Z}$ denotes the circle endowed with the topology induced by the arc-length metric *d*, and the parameter family of maps defined as

$$\begin{aligned}T_a(x):=T(x+a\pmod {1}).\end{aligned}$$

We consider random iteration of maps from this family

$$\begin{aligned} x_n = T^n_\omega (x_0):= T_{\omega _{n-1}} \circ \cdots \circ T_{\omega _1} \circ T_{\omega _0} (x_0) \end{aligned}$$

where $\omega := (\omega _i)_{i\in \mathbb {N}}$ and $\omega _i$ is drawn randomly (i.i.d.) from $[-\vartheta ,\vartheta ]$ with uniform measure $\textrm{Leb}/(2\vartheta )$. We denote the corresponding sequence space

$$\begin{aligned} \Sigma _\vartheta :=[-\vartheta ,\vartheta ]^\mathbb {N}. \end{aligned}$$

with corresponding product measure $\mathbb {P}$.

Our results require certain mild hypotheses, Hypothesis ()(H1)–()(H5), which we proceed to sketch with reference to Sect. 2 for details.

We assume that *T* and $\vartheta$ are such that the random dynamical system has a unique stationary measure $\mu$ with smooth and everywhere positive density. As a result, the random dynamical system has a single Lyapunov exponent

$$\begin{aligned} \lambda = \frac{1}{2\vartheta } \int _\mathbb {T}\int _{[-\vartheta ,\vartheta ]} \ln \left( DT_\omega (x)\right) \, d\textrm{Leb}(\omega ) d\mu (x), \end{aligned}$$

which can be negative, zero, or positive, depending on *T* and $\vartheta$. When we consider the case where the random dynamical system depends smoothly on an additional parameter, the Lyapunov exponent also varies smoothly with this parameter (due to our hypotheses). Then, a transition to chaos corresponds to the Lyapunov exponent traversing zero from below.

We consider the two-point motion to gain insights into the dynamics:

$$\begin{aligned} (x_n,y_n) = (T^{(2)}_\omega )^n (x_0,y_0) :=\left( T^n_\omega (x_0), T^n_\omega (y_0) \right) . \end{aligned}$$

Stationary measures for the two-point random dynamical system provide information well beyond stationary measure for (the one-point dynamics of) $T_\omega$. Note that the stationary measure for $T_\omega$ is also a stationary measure of the two-point motion, supported on the diagonal

$$\begin{aligned} \Delta :=\left\{ (x,x) \in \mathbb {T}^2 \;; \; x \in \mathbb {T} \right\} . \end{aligned}$$

We are primarily interested in stationary measures with support outside of $\Delta$, providing detail on the comparison between orbits with different initial conditions. The $\varepsilon$-neighbourhood of the diagonal is denoted as

$$\begin{aligned} \Delta _\varepsilon :=\left\{ (x,y) \in \mathbb {T}^2 \;; \; d(x,y) < \varepsilon \right\} . \end{aligned}$$

Our main result concerns the following trichotomy in phenomenology from a topological and measure theoretic point of view:

#### Theorem 1.1

(Topological random dynamics). Let $T_\omega$ be a random dynamical system satisfying hypotheses (H1) to (H5), $\lambda$ be its Lyapunov exponent, and let $T^{(2)}_\omega$ be the corresponding two-point random dynamical system. Then,

1. **Synchronisation:** if $\lambda <0$, for all $x,y \in \mathbb {T}$,
  $$\begin{aligned} \lim _{n\rightarrow \infty } d\left( T^n_\omega (x),T^n_\omega (y)\right) = 0,~\mathbb {P}-\mathrm {a.s.} \end{aligned}$$
2. **Intermittency:** if $\lambda =0$, for all $(x,y) \in \mathbb {T}^2\setminus \Delta$,
  $$\begin{aligned} \lim _{n\rightarrow \infty } \frac{1}{n}\sum _{i=0}^{n-1} d\left( T^i_\omega (x),T^i_\omega (y)\right) = 0 ~\text {and}~ \limsup _{n\rightarrow \infty }d\left( T^n_\omega (x), T^n_\omega (y)\right) > 0,~\mathbb {P}-\mathrm {a.s.} \end{aligned}$$
3. **Chaos:** if $\lambda > 0$, for all $(x,y) \in \mathbb {T}^2\setminus \Delta$,
  $$\begin{aligned} \lim _{n\rightarrow \infty }\frac{1}{n}\sum _{i=0}^{n-1} d \left( T^i_\omega (x), T^i_\omega (y)\right) > 0 ~\text {and}~ \liminf _{n\rightarrow \infty }d\left( T^n_\omega (x),T^n_\omega (y)\right) = 0,~\mathbb {P}-\mathrm {a.s.} \end{aligned}$$

This result mirrors those in [41], obtained for special random affine circle maps. The most interesting aspect concerns the existence of intermittency in part (2). The existence of synchronisation in the presence of negative Lyapunov exponent has already been well-studied, see e.g. [56], and the properties highlighted under part (3) also align with existing insights. The characterization of intermittency in part (2) is reminiscent of synchronisation on average [37] and that of chaos in part (3) of Li-Yorke chaos [18, 48]. These characterisations are not exhaustive. For instance, recently the positive Lyapunov exponent regime has been associated with the existence of so-called random horse-shoes [44, 45].

The proof of Theorem 1.1, which uses techniques introduced in Sects. 2 and 3, is given in Sect. 4. The results for $\lambda <0,\lambda =0$ and $\lambda >0$ are presented separately in Propositions 4.8, 4.3 and 4.6, respectively.

#### Theorem 1.2

(Stationary measures). Let $T_\omega$ be as in Theorem 1.1, and let $\mu$ denote its unique stationary (probability) measure.

Then,

1. If $\lambda < 0$, $\mu$ on $\Delta$ is the unique stationary measure for $T^{(2)}_\omega$.
2. If $\lambda =0$, $\mu$ on $\Delta$ is the unique stationary probability measure for $T^{(2)}_\omega$. In addition, $T^{(2)}_\omega$ admits an infinite stationary (Radon) measure $\mu ^{(2)}$ on $\mathbb {T}^2\setminus \Delta$, which has full support. Moreover, for each such measure there exist $\alpha , \beta \in (0,\infty )$ such that
  $$\begin{aligned} \alpha \le \liminf _{\varepsilon \rightarrow 0} \frac{\mu ^{(2)}(\mathbb {T}^2\setminus \Delta _\varepsilon )}{-\ln (\varepsilon )} \le \limsup _{\varepsilon \rightarrow 0} \frac{ \mu ^{(2)}(\mathbb {T}^2\setminus \Delta _\varepsilon )}{-\ln (\varepsilon )} \le \beta . \end{aligned}$$
3. If $\lambda >0$, in addition to the unique stationary measure $\mu$ on $\Delta$, $T^{(2)}_\omega$ admits a stationary probability measure $\mu ^{(2)}$ on $\mathbb {T}^2\setminus \Delta$, which has full support. Moreover, if the non-zero root $\gamma <0$ of the moment Lyapunov function^1^ associated with $T_\omega$ is also larger than $-\frac{1}{2}$, then for each such measure there exists $\alpha , \beta \in (0,\infty )$, such that
  $$\begin{aligned} \alpha \le \liminf _{\varepsilon \rightarrow 0} \frac{\mu ^{(2)}(\Delta _\varepsilon )}{\varepsilon ^{-\gamma }} \le \limsup _{\varepsilon \rightarrow 0} \frac{\mu ^{(2)}(\Delta _\varepsilon )}{\varepsilon ^{-\gamma }} \le \beta . \end{aligned}$$

As in Theorem 1.1, the most interesting aspect of this result is the intermittent case in part (2), where we find that ergodic invariant measures off the diagonal are infinite, together with the estimate on how such measures grow near the diagonal.

For an ergodic stationary measure in case of a positive Lyapunov exponent, the estimates on $\mu ^{(2)}(\Delta _\varepsilon )$ determine bounds on the frequency with which orbits of the random map are within distance $\varepsilon$ of each other, by Birkhoff’s ergodic theorem. The asymptotics of the stationary invariant measure near the diagonal in the positive Lyapunov exponent scenario (in part (3)) relies on the condition that $\gamma \in (-\frac{1}{2},0)$. If $\gamma <-\frac{1}{2}$, then the asymptotics may be different due to points being mapped more frequently close to the diagonal, see Proposition 5.5.

The construction of stationary measures off the diagonal for the two-point motion in parts (2) and (3) makes use of an inducing scheme with randomized return times, in order to apply a Krylov–Bogolyubov type argument. It should be noted that such reasoning does not provide uniqueness of stationary measures.

The heart of the proof of the above theorem is presented in Sect. 5, with arguments relying on estimates from Sects. 3 and 4. The existence of the stationary measure on $\Delta$ follows from Proposition 2.1. For $\lambda <0$, Theorem 1.1 (1) directly implies the unique stationary measure in Theorem 1.2 (1). The results for $\lambda =0$ and $\lambda > 0$ follow from Propositions 5.1, 5.4 and 5.5.

We proceed to illustrate our results in an example.

**Fig. 1:**
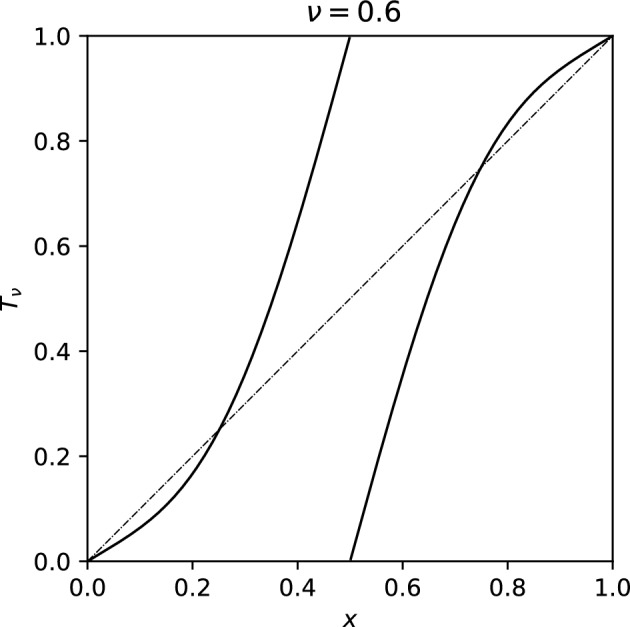
The graph of $T_{\nu }$ (1.2) at $\nu =0.6$

#### Example 1.3

We consider a one-parameter family of random circle endomorphism

$$\begin{aligned} x_n=T_{\nu ,\omega }^n(x_0):= T_{\nu ,\omega _{n-1}} \circ \cdots \circ T_{\nu ,\omega _1} \circ T_{\nu ,\omega _0} (x_0) \end{aligned}$$

with $\omega _i$ drawn i.i.d. from the uniform distribution on a subinterval of the circle $[-\vartheta ,\vartheta ]$, and

1.1 $$\begin{aligned} T_{\nu ,a}(x) := T_\nu (x +a \pmod 1), \end{aligned}$$

with

1.2 $$\begin{aligned} T_\nu (x) := \int _0^x \nu + 140 (2-\nu ) t^3(1-t)^3 dt \pmod {1}. \end{aligned}$$

**Fig. 2:**
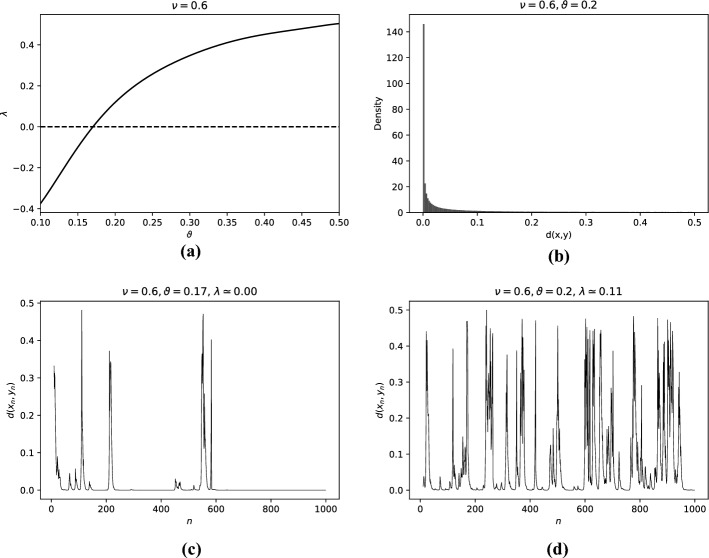
Illustration of aspects of the dynamics in Example 1.3 regarding the family of random maps $T_{\nu ,\omega }$ with $\nu = 0.6$ **(a)** Numerical approximation of the Lyapunov exponent for fixed $\nu = 0.6$ and varying $\vartheta$. The Lyapunov exponent increases monotonically with the noise level, from negative to positive (noise-induced chaos). **(b)** Normalized distribution of distance between two orbits ($10^6$ iterations) with different initial conditions and the same noise realisation, consistent with Theorem 1.2 (). **(c)** Example of a time series of the distance between two orbits for $\vartheta = 0.17$, when $\lambda \approx 0$, close to intermittency. **(d)** Example of a time series of the distance between two orbits for $\vartheta = 0.2$, when $\lambda \approx 0.11$, in the chaotic regime

Note that $T_\nu (0)=0$ and $DT_\nu (0) = \nu$. In Fig. 1 we sketch the graph of $T_\nu$ for $\nu = 0.6$. Fig. 2 depicts results of numerical experiments for this random family. We remark that it is possible to determine Lyapunov exponents and the Lyapunov moment functions rigorously [19, 35].

For parameter ranges discussed in this example $T_{\nu ,\omega }$ satisfies the Hypotheses (H1) to (H5), see Example 2.6.

We first consider the behaviour of the Lyapunov exponent at a fixed value of $\nu = 0.6$ with varying $\vartheta$. In Fig. 2a, we observe noise-induced chaos, as the Lyapunov exponent increases monotonically from $-0.4$ to 0.45 with increasing $\theta \in [0.1,0.5]$. In this interval, the noise is large enough to ensure the existence of a unique stationary measure for $T_{0.6,\omega }$ on $\mathbb {T}$.

The dynamics of the two-point motions is illustrated by examples of time series for two different values of $\vartheta$ in Fig. 2c ($\theta =0.17$, close to intermittency) and Fig. 2d ($\theta =0.2$, chaos), displaying qualitative differences consistent with Theorem 1.1.

Figure 2b shows the distribution of the two-point distance for an example time-series, displaying inverse-power-law behavior near zero, as asserted in Theorem 1.2.

**Fig. 3:**
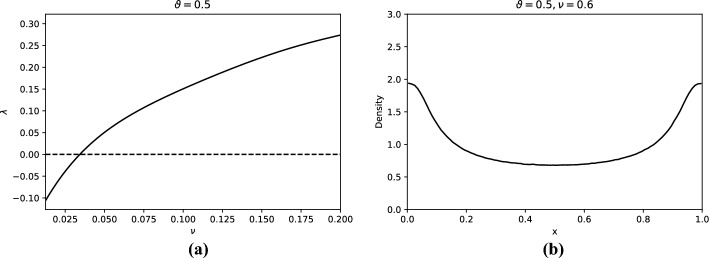
Illustration of aspects of the dynamics of Example 1.3 regarding the family of random maps $T_{\nu ,\omega }$, with $\vartheta = 0.5$ and varying $\nu$. **(a)** Numerical approximation of the Lyapunov exponent for fixed $\vartheta = 0.5$ and varying $\nu$. The Lyapunov exponent increases monotonically with $\nu$, from negative to positive. **(b)** Numerical approximation of the stationary density, with $\nu =0.6$ and $\vartheta = 0.5$

Rather than varying the noise level for fixed parameter $\nu$, one may also consider varying $\nu$ at fixed noise level. Fixing the noise level at $\theta =0.5$ (full noise), yields one-point motion orbits to be random i.i.d. sample from the stationary measure $\mu = \left( T_\nu \right) _* (\textrm{Leb})$, see Fig. 3b.

We include this example as it highlights the difference between the one- and two-point motion. In particular, at full noise, the one-point dynamics is essentially a full shift while the dynamics may be synchronising or chaotic. Indeed, varying $\nu$ between 0.025 and 0.2 we observe (in Fig. 3a ) a monotonically increasing Lyapunov exponent going from negative (synchronisation) to positive (chaos).

We revisit the full noise case in the context of this example, in Examples 3.9 and 3.12, where this assumption leads to simplified statements.

### Context

In this paper we have established a link between intermittency (of the two-point motion) and the transition to chaos in random dynamical systems. In the deterministic setting, intermittency has been recognized as a hallmark of only one of several routes to chaos [62].

The link between intermittency and infinite ergodic measures has been previously observed for the one-point motion, concerning the behaviour of single trajectories. First, this was established for deterministic dynamics near non-hyperbolic fixed points [50, 59, 65]. Later, this was extended to intermittent dynamics near unstable invariant manifolds, also know as on-off intermittency [38, 61]. In random maps on bounded state spaces with common neutral fixed points, intermittency and infinite ergodic measures have been established in the presence of neutral fixed points [6, 7] and on-average neutral fixed points [58, 66]. See [1, 4, 36] for case studies of random logistic maps with zero Lyapunov exponents. Random dynamical systems on unbounded state spaces with zero Lyapunov exponent may also yield infinite stationary measures [5, 21–23, 31]. For specific results on synchronisation on average (related to our notion of intermittency) in random maps, see also [37, 52].

While the majority of research in ergodic theory of dynamical systems focuses on one-point motions, two-point motions are central to this paper. Crucially, our results on intermittency and infinite ergodic measures of the two-point motion of random maps with zero Lyapunov exponents do not imply intermittency and infinite ergodic measures for the one-point motion. In particular, the one-point motion of the random maps discussed in this paper do not display intermittency nor infinite ergodic measures.

The importance of two-point motions, or more general *n*-point motions, has been recognized in the study of stochastic differential equations, see for instance [43], and in particular by Ledrappier and Young [46, 47] and Baxendale and Stroock [11, 13] focusing on different Lyapunov exponent regimes. More recently, their importance has been recognized in stochastic fluid dynamics [14–17, 20].

Synchronisation in random dynamical systems constitutes the most elementary type of dynamical behaviour, and has been studied extensively [34, 40, 53]. In particular, it has been established in the negative Lyapunov exponent regime that synchronisation is equivalent to a mild contractability condition [56]. Specifically, random circle homeomorphism have been discussed in [2, 42, 51, 68].

There is also an interest in iterated function systems [9] where the random parameter is chosen from a finite set, see also [41] for a result in the direction of this paper. Our methods do not cover this setting, as we use absolute continuity of the noise to obtain spectral gap for a Koopman operator on a space of continuous functions. A possible strategy for discrete noise models might be to consider smaller function spaces.

There are various problems beyond this paper that deserve further attention. First of all, we expect our results to extend to higher-dimensional settings and allow for critical points (see [35] for a recent numerical study in this direction). Furthermore, it is natural to study parametrized families of random dynamical systems and consider the dependence on the parameter of, in particular, Lyapunov moment function and stationary measures of the two-point motion near zero Lyapunov exponent (see [27] for a recent study in the case of random matrix products). Finally, it would be of interest to obtain finer statistical information about the two-point dynamics in the presence of infinite stationary measures.

### Organization of the paper

This paper is divided into six sections. In Sect. 2, we discuss the underlying hypotheses of this paper and provide a summary of the strategy of the proof. In Sect. 3, the technical machinery involving Koopman operators is developed, which is required in the subsequent sections. Section 4 is dedicated to the proof of Theorem 1.1, where the three different scenarios with zero, positive, and negative Lyapunov exponent are treated, respectively in Sects. 4.1, 4.2, and 4.3. The construction of stationary measures and the derivation of their properties, leading to Theorem 1.2, are contained in Sect. 5.

## Setting and Strategy of the Proof

This section introduces the hypotheses on the system and includes a summary of the strategy of the proof. We start with a circle endomorphism $T: \mathbb {T}\rightarrow \mathbb {T}$ with the following property.

*T* is a circle endomorphism in $\mathcal {C}^2(\mathbb T)$, of degree two with derivative positively bounded from above and below; there exist positive real numbers $a_1< a_2$, such that

{\bf H1 $$\begin{aligned}&a_1<DT (x)<a_2 \end{aligned}$$

for all $$x \in \mathbb T$$.

Note that every point has exactly two inverse images under *T*. Degree two is not essential in this paper, higher degree works the same with minor modifications. Define

2.1 $$\begin{aligned}  &   R_{\min } :=\max \left\{ r \; ; \; a_1 d(x,y) \le d\left( T(x),T(y)\right) \right. \nonumber \\  &   \quad \left. \le a_2 d(x,y) \text { for all } x,y \in \mathbb {T}\text { with } d(x,y)\le r \right\} . \end{aligned}$$

In particular $T(x) \ne T(y)$ if $d(x,y) \le R_{\min }$.

For $$\omega \in \mathbb {T}$$ write

$$\begin{aligned} T_\omega (x)&= T(x + \omega \pmod 1) \end{aligned}$$

for the circle endomorphism obtained by composing *T* with a translation. We take a random process $\omega _i$, $i \in \mathbb {N}$, where the $\omega _i$ are independently drawn from a uniform distribution on

$$\begin{aligned} \Omega _\vartheta :=[-\vartheta ,\vartheta ]. \end{aligned}$$

This yields a random dynamical system

2.2 $$\begin{aligned} x_{n+1}&= T_{\omega _n} (x_n) \end{aligned}$$

for an initial point $x_0 \in \mathbb {T}$.

Let the sequence space

$$\begin{aligned} \Sigma _\vartheta :=\Omega _\vartheta ^\mathbb {N} \end{aligned}$$

be endowed with the product topology. We write $\omega = (\omega _i)_{i\in \mathbb {N}}$ for points in ${\Sigma _\vartheta }$. Cylinders are sets $[A_0,A_1\ldots ,A_k] = \{ \omega \in {\Sigma _\vartheta } \;; \; \omega _i \in A_i, 0\le i \le k\}$ for Borel sets $A_i \subset \mathbb {T}$. Cylinders are the basis for the product topology. Given the normalized Lebesgue measure $\textrm{Leb}/(2\vartheta )$ on $\Omega _\vartheta$, write $\mathbb {P}$ for the corresponding product measure on ${\Sigma _\vartheta }$. For a function *X* on $\Sigma _\vartheta$ we use common notation such as

$$\begin{aligned} \mathbb {E} \left[ X \right]&:=\int _{\Sigma _\vartheta } X(\omega )\, d\mathbb {P}(\omega ). \end{aligned}$$

By identifying $\Omega _\vartheta$ with the cylinder $[\Omega _\vartheta ]$ we can also use $\mathbb {P}$ for normalized Lebesgue measure $\textrm{Leb}/(2\vartheta )$ on $\Omega _\vartheta$. So, with a slight abuse of notation, if a function $\omega \mapsto X(\omega )$ depends on a single symbol $\omega \in \Omega _\vartheta$, we write $\mathbb {E} \left[ X \right] = \int _{\Omega _\vartheta } X(\omega )\, d\mathbb {P}(\omega )$. Recall that a measure $\mu$ on $\mathbb {T}$ is called a stationary measure if

$$\begin{aligned} \int _{\Sigma _\vartheta } \mu \left( \left( T_\omega \right) ^{-1} (A) \right) \, d\mathbb {P} (\omega ) = \mu (A), \end{aligned}$$

for any (Borel) measurable set $A \subset \mathbb {T}$.

The skew product map $\Theta : {\Sigma _\vartheta } \times \mathbb {T} \rightarrow {\Sigma _\vartheta } \times \mathbb {T}$ is defined by

$$\begin{aligned} \Theta (\omega ,x) :=(\sigma \omega , T_{\omega _0} (x)). \end{aligned}$$

Here $\sigma$ is the left shift operator $\sigma \omega :=(\omega _{i+1})_{i\in \mathbb {N}}$. With a slight abuse of notation we write

$$\begin{aligned} T^n_\omega (x) :=T_{\omega _{n-1}} \circ \cdots \circ T_{\omega _0} (x) \end{aligned}$$

for iterates.

We compares two different trajectories by studying the random dynamical system. For $\omega \in \Sigma _\vartheta$, the two-point map $(x,y) \mapsto T^{(2)}_\omega (x,y)$ on $\mathbb {T}^2$ is the product

$$\begin{aligned} (x,y) \mapsto (T_\omega (x), T_\omega (y)). \end{aligned}$$

This yields the random dynamical system

2.3 $$\begin{aligned} (x_{n+1},y_{n+1})&= T^{(2)}_{\omega _n} (x_n,y_n). \end{aligned}$$

The two-point skew product map $\Theta ^{(2)}: {\Sigma _\vartheta } \times \mathbb {T}^2 \rightarrow {\Sigma _\vartheta } \times \mathbb {T}^2$ is denoted by

$$\begin{aligned} \Theta ^{(2)} (\omega ,x,y) = (\sigma \omega , T^{(2)}_\omega (x,y)). \end{aligned}$$

A measure $\mu ^{(2)}$ on $\mathbb {T}^2$ is a stationary measure of the random dynamical system $T^{(2)}_\omega$ on $\mathbb {T}^2$ if

$$\begin{aligned} \int _{\Sigma _\vartheta } \mu ^{(2)} \left( \left( T^{(2)}_\omega \right) ^{-1} (A) \right) \, d\mathbb {P} (\omega ) = \mu ^{(2)}(A), \end{aligned}$$

for any (Borel) measurable set $A \subset \mathbb {T}^2$.

### Hypotheses

We focus on random circle endomorphisms whose trajectories are not confined to subintervals of the circle but spread over the entire circle.

{\bf H2 $$\begin{aligned} \hbox {There is }k>0\hbox { so that for any }x,y \in \mathbb {T},\hbox { there is } \omega \in {\Sigma _\vartheta }\hbox { so that }T^k_\omega (x) = y. \end{aligned}$$

This hypothesis in combination with absolute continuous noise guarantees the existence of a unique absolutely continuous stationary measure of full support for the one-point motion, but also has further applications that are used throughout the paper.

#### Proposition 2.1

Suppose the random dynamical system described by (2.2) with $\omega _n$ i.i.d. picked from a uniform distribution for $[-\vartheta ,\vartheta ]$, adheres to Hypotheses (H1), (H2).

Then the random dynamical system admits an absolutely continuous stationary measure $\mu$ with full support and smooth density.

The proof is omitted and be obtained from standard arguments using Perron-Frobenius operators. A similar setup is in [8], to which we refer for details.

The following assumption provides a local source for contraction. This is needed to achieve a negative or zero Lyapunov exponent.

{\bf H3 $$\begin{aligned} \hbox {The map }T\hbox { has a hyperbolic attracting periodic orbit.} \end{aligned}$$

We must avoid that points in $\mathbb {T}^2 \setminus \Delta$ are mapped into $\Delta$ by $T^{(2)}_a$ with positive probability, and more generally we must control and bound the probability that points are mapped very close to the diagonal by the two-point maps $T^{(2)}_a$. The following hypothesis provides us with an assumption to prevent this.

{\bf H4 $$\begin{aligned}&\hbox {For every }(x,y) \in \mathbb {T}^2\setminus \Delta ,\hbox { the curve }a \mapsto (T_a (x), T_a (y))\hbox { in }\mathbb {T}^2\\&\quad \hbox { intersects the diagonal} \Delta \hbox { transversely or with at most a single quadratic tangency.} \end{aligned}$$

Hypothesis (H4) is a generic condition. For convenience we formulate the following lemma with the parameter *a* in $T_a$ from the whole circle $\mathbb {T}$; taking *a* from a larger interval only shrinks the set of circle endomorphisms that satisfy Hypothesis (H4).

#### Lemma 2.2

There is an open and dense set of degree two increasing circle endomorphisms $T: \mathbb {T} \rightarrow \mathbb {T}$ in the $\mathcal C^2$ topology so that the corresponding random system $T_a$ with $a \in \mathbb {T}$ satisfies Hypothesis (H4).

#### Proof

To study intersections of the curve $a \mapsto (T_a (x), T_a (y))$ with $\Delta$ we only need to consider nearby points $T(x+a), T(y+a)$. We may thus write $T(x+a) - T(y+a) = 0$ instead of $d(T(x+a),T(y+a))=0$. Write $T(x+a) = T(y+a)$ as $T(b+s) = T(b)$ with $b = x+a$ and $b+s = y+a$, that is, $s = y-x$. We are thus looking for zeros of the family of functions

$$\begin{aligned} f_s (b) :=T(b+s) - T(b), \end{aligned}$$

with $b \in \mathbb {T}$. Note that zero’s of $f_s$ for $b+s \ne b$ can only occur for *s* away from zero. The hypothesis can be rephrased as stating that the graph of the function $f_s$ has only transverse intersections with zero, or at most one quadratic tangency to the graph of the zero function for isolated values of *s*. As in classical bifurcation theory applied to bifurcations of equilibria in one-parameter families [24, 57, 64], for an open and dense set of degree two increasing circle endomorphisms, a zero is either transverse or with a quadratic tangency, and there is at most one zero with a quadratic tangency (a quadratic tangency corresponds to a saddle-node bifurcation, for generic families one finds at most one saddle-node bifurcation at a parameter value).

The integrability statement in the following lemma is a consequence of Hypothesis (H4), and results in an estimate that is required in later arguments (Propositions 4.6, 5.4 and 5.5).

#### Lemma 2.3

For all $R>0$ there exists $C_1>0$ with

2.4 $$\begin{aligned} \mathbb {E} \left[ -\ln (d(T_\omega ^{(2)}(x,y))) \right] < C_1, \end{aligned}$$

for all $$(x,y) \in \mathbb {T}^2 \setminus \Delta _{R}$$.

#### Proof

Let *w*(*x*, *y*) denote the signed distance of $x,y\in \mathbb {T}$, defined as $w(x,y) = \left( (y - x + \frac{1}{2}) \pmod 1 \right) - \frac{1}{2}$. Consider the real valued function

$$\begin{aligned}a \mapsto f(a) :=w\left( T^{(2)}_a (x,y) \right) \end{aligned}$$

on $\mathbb {T}$. Hypothesis (H4) implies that (see also the proof of Lemma 2.2) for $(x,y) \in \mathbb {T}^2 {\setminus } \Delta _\delta$, there exist $\delta>0, C>0$ so that

$$\begin{aligned} \left| D^2 f (a) \right|&\ge C \end{aligned}$$

whenever $$|f(a)| < \delta $$ and $$\left| Df(a) \right| < \delta $$.

If $I \subset \mathbb {T}$ is an interval so that for $a \in I$, $|f(a)| < \delta$ and $\left| D f(a) \right| \ge \delta$, then $\int _I -\ln \left( |f(a)| \right) \, da$ is bounded by some $C_\delta >0$.

To prove the lemma it remains to consider *f* on an interval $$J \subset \mathbb {T}$$ so that $$|f(a)| < 2 \delta $$ for $$a \in J$$ and *J* contains a point $$d \in J$$ with $$Df(d) = 0$$. If $$f(d) = 0$$ and $$Df(d) = 0$$, we have a bound $$0< c (a-d)^2 < |f(a)|$$ for some $$c > 0$$. The same bound applies if $$Df(d) = 0$$ and *f* is never zero on *J*. The possibility that is left is where *f* has two zeros $$f(d_1) = f(d_2) = 0$$ for nearby points $$d_1,d_2$$ on different sides of *d*. Then a bound $$0< c |(a-d_1) (a-d_2)| < |f(a)|$$ for some $$c > 0$$ holds. In all cases *c* is uniformly bounded away from 0. As the logarithms of $$c (a-d)^2$$ and $$ c |(a-d_1) (a-d_2)|$$ are integrable, we find that $$\int _J -\ln \left( |f(a)| \right) \, da$$ is bounded by some $$C_\delta >0$$. Noting that $$-\ln ( |f(a)| )$$ is bounded if $$|f(a)| > \delta $$, proves the lemma.

We make a final assumption that will be used to prove full support of stationary measures for the two-point motion. The random two-point system maps a point (*x*, *y*) to a curve $a \mapsto (T_a (x),T_a(y))$ in $\mathbb {T}^2$. For the composition of two iterates, we have

2.5 $$\begin{aligned} H_{a_0,a_1}(x,y)&:=\textrm{det}\, \left( \frac{\partial }{\partial (a_0,a_1)} T^{(2)}_{a_1} \circ T^{(2)}_{a_0} (x,y) \right) \nonumber \\&= DT_{a_1} ( T_{a_0}(x)) DT_{a_1} ( T_{a_0}(y)) \left( DT_{a_0} (x) - DT_{a_0} (y) \right) . \end{aligned}$$

Consider now the following hypothesis.

{\bf H5 $$\begin{aligned} \hbox {For each }(x,y) \in \mathbb {T}^2 \setminus \Delta ,\hbox { there are } a_0,a_1 \in (-\vartheta ,\vartheta )\hbox { so that }H_{a_0,a_1}(x,y) \ne 0. \end{aligned}$$

This condition implies that for each $(x,y) \in \mathbb {T}^2 {\setminus } \Delta$, the image of

$$\begin{aligned} (a_0,a_1) \mapsto T^{(2)}_{a_1} \circ T^{(2)}_{a_0} (x,y), \end{aligned}$$

with $a_0,a_1 \in [-\vartheta ,\vartheta ]$, contains an open set.

Recall that a map $F: X \rightarrow X$ on a topological space *X* is called topologically exact if for any open $U \subset X$, there is $n \in \mathbb {N}$ so that $F^n (U) = X$.

#### Lemma 2.4

The skew product systems $\Theta : {\Sigma _\vartheta } \times \mathbb {T} \rightarrow {\Sigma _\vartheta } \times \mathbb {T}$ and $\Theta ^{(2)}: {\Sigma _\vartheta } \times \mathbb {T}^2 \rightarrow {\Sigma _\vartheta } \times \mathbb {T}^2$ are topologically exact.

#### Proof

We first consider $\Theta$ and we start with the following statement: for any open interval $I \subset \mathbb {T}$ there is $\omega \in \Sigma _\vartheta$ and $k\in \mathbb {N}$ so that $T_\omega ^k (I) = \mathbb {T}$. The reasoning will also show that there is a cylinder $D \subset \Sigma _\vartheta$ that contains $\omega$, so that $T_\zeta ^k (I) = \mathbb {T}$ for any $\zeta \in D$.

Given the interval *I*, take $x \in I$. Let $y \in \mathbb {T}$ be a periodic point in an uncountable transitive hyperbolic repelling set $\Lambda$ of a map $T_a$ [54]. Write *k* for the period of *y*. We claim that given an open neighborhood *V* of *y*, there is an iterate $T^{kn}_a$ that maps *V* onto all of $\mathbb {T}$. Suppose not. If *V* is small then $T_a^k$ is a diffeomorphism between *V* and $T_a^k(V)$. Keep iterating until either some iterate $T_a^{kn}$ no longer defines a diffeomorphism between $T_a^{k(n-1)} (V)$ and $T_a^{kn}(V)$ (then $T_a^{kn} (V)$ covers $\mathbb {T}$, or this never happens. In the latter case, $\cup _n T^{kn}_a (V)$ is an interval and $T_a^k$ is a diffeomorphism on it. This is not possible since *y* is accumulated by repelling periodic points of different periods.

By Hypothesis (H2) there is $\varsigma \in \Sigma _\vartheta$ and $m \in \mathbb {N}$ so that $T_\varsigma ^m (x) = y$. Hence there is $n \in \mathbb {N}$ so that $T^{kn}_a \circ T^m_\varsigma$ maps *I* onto $\mathbb {T}$. By continuity the same applies to $T^{m+kn}_\nu$ for $\nu$ in a suitable cylinder *D* that contains $\varsigma _0\cdots \varsigma _{m-1} a \cdots a$ (with *nk* times *a*). This proves the statement with which we started.

To conclude the proof of topological exactness of $\Theta$, let $U\subset \Sigma _\vartheta \times \mathbb {T}$ be an open set. By shrinking *U* we may assume that *U* is a product set $U = C \times J$ of a cylinder *C* and an open interval *J*. There is an iterate $\Theta ^j (U)$ that contains an open set $\Sigma _\vartheta \times I$ for an open interval $I \subset \mathbb {T}$. Now use the above statement to establish that a further iterate covers $\Sigma _\vartheta \times \mathbb {T}$.

Next we consider $\Theta ^{(2)}$. Take an open set $U \subset \Sigma _\vartheta \times \mathbb {T}^2$. By shrinking *U* we may assume that *U* is a product set $U = C \times I \times J$ of a cylinder *C* and open intervals *I*, *J*.

By the above reasoning, there is $l \in \mathbb {N}$ and $\omega \in C$ so that $\left( T^{(2)}_\omega \right) ^l (I \times J)$ contains $\mathbb {T} \times K$ for an open interval $K \subset \mathbb {T}$. Applying the above reasoning again, there is $l \in \mathbb {N}$ so that $\left( T^{(2)}_\omega \right) ^l (I\times J)$ equals $\mathbb {T}^2$. The proof of topological exactness of $\Theta ^{(2)}$ is concluded as above.

Hypotheses (H3), (H5) and Lemma 2.4 show that $\mathbb P$-almost surely all orbits of the two point motion get arbitrarily close to the diagonal $\Delta$. The following lemma formalizes this.

#### Lemma 2.5

For all $\varepsilon >0$, there exists a $N\in \mathbb {N}$, $C>0$ such that for all $(x,y) \in \mathbb {T}^2$, we have

$$\begin{aligned} \mathbb {P} \left( \left\{ \omega \in \Sigma _\vartheta \;; \; d(T_\omega ^i(x),T^i_\omega (y))<\varepsilon \text{ for } \text{ some } 0\le i \le N \right\} \right) > C. \end{aligned}$$

#### Proof

Take $(x,y) \in \mathbb {T}^2 \setminus \Delta _\varepsilon$. By Hypothesis (H5) we find that $\omega \mapsto \left( T^{(2)}_\omega \right) ^2 (x,y)$ contains an open product set $I_x\times I_y \subset \mathbb {T}^2$. By compactness we find $\delta >0$ so that $I_x$ and $I_y$ have diameter at least $\delta$, uniformly in $x,y \in \mathbb {T}^2 \setminus \Delta _\varepsilon$. The argument of Lemma 2.4 shows that for any $(x,y) \in \mathbb {T}^2 {\setminus } \Delta _\varepsilon$ there is an open neighborhood *U* of (*x*, *y*) and a positive integer *N* so that $\left( T^{(2)}_\omega \right) ^N(x',y')$ covers $\mathbb {T}^2$ for any $(x',y') \in U$. By compactness *N* is bounded uniformly in $(x,y) \in \mathbb {T}^2 {\setminus } \Delta _\varepsilon$. This implies the lemma.

#### Example 2.6

We continue from Example 1.3, giving concise justifications that the required hypotheses hold. Hypotheses (H1) and (H3) are trivially satisfied. Hypothesis (H2) is obvious when $\vartheta = 0.5$, and can be checked via a simulation or some naive bounds. The main obstruction to Hypothesis (H2) would be that the noise is too small to let an orbit escape from a neighborhood of the stable fixed point.

Regarding Hypothesis (H4), observe that there is exactly one point $y \in \mathbb {T}$ that has two preimages $x_1, x_2 \in \mathbb {T}$ with

$$\begin{aligned} DT(x_1) = DT(x_2). \end{aligned}$$

At this point *y*, the second derivatives $DT(x_1)$ and $DT(x_2)$ have opposite signs. The curvature of the curve $a \mapsto (T_a (x_1), T_a (x_2))$ at the point of tangency with $\Delta$ is therefore nonzero, and the tangency is quadratic.

Finally, by Eq. (2.5), and the fact that for all $(x,y) \in \mathbb {T}^2{\setminus } \Delta$, there exists an $a_0 \in [-\vartheta ,\vartheta ]$ such that $DT_{a_0}(x) - DT_{a_0}(y) \ne 0$, Hypothesis (H5) follows. $\square$

### Strategy of the proof

This section gives a helicopter view on the reasoning in Sects. 3 and 4 that leads to Theorem 1.1 on dynamics of the random two-point maps. For the study of orbits of the random two-point maps it is crucial to understand the duration of trajectories of two nearby points staying close to each other. In terms of the two-point motion this is the duration of trajectories of the two-point motion staying close to the diagonal. Suppose $x_0,y_0 \in \mathbb {T}$ are close, so that $d_0 = d(x_0,y_0)$ is close to zero. With $x_n = T^n_\omega (x_0)$, $y_n = T^n_\omega (y_0)$ and $d_n = d(x_n,y_n)$, we find that as long as $d_n$ is small,

2.6 $$\begin{aligned} d_{n+1}&\approx DT_{\omega _n} (x_n) d_n. \end{aligned}$$

Taking logarithms $u_n = \ln (d_n)$ this reads

2.7 $$\begin{aligned} u_{n+1}&\approx u_n + \ln \left( DT_{\omega _n}(x_n) \right) , \end{aligned}$$

which is a random walk driven by the one-point motion $x_{n+1} = T_{\omega _n} (x_n)$.

In the special case where $x_n$ is identically and independently distributed (see Example 1.3, with $\vartheta = 0.5$), the approximation is a random walk with i.i.d. steps. But this is not true in general. We derive estimates for the duration of passages near the diagonal by adapting reasoning in [11, 13] for continuous time settings to our discrete time setting. We base our analysis on properties of the Koopman operator for the two-point system.

For the one-point motion, the annealed Koopman operator *P* acting on a real valued function $\phi$ on $\mathbb {T}$ is defined as

$$\begin{aligned} P \phi (x)&= \mathbb {E} \left[ \phi (T_\omega (x) ) \right] . \end{aligned}$$

Here $\mathbb {E}$ stands for an expectation over $\omega \in \Sigma _\vartheta$. Analogously the annealed two-point Koopman operator $P_{(2)}$ acting on a real valued function $\phi$ on $\mathbb {T}^2\setminus \Delta$ is defined as

$$\begin{aligned} P_{(2)}\phi (x,y) = \mathbb {E} \left[ \phi (T_\omega ^{(2)}(x,y))\right] . \end{aligned}$$

To estimate stopping times for trajectories of the two-point motion from strips $\Delta _\delta \setminus \Delta _\varepsilon$ near the diagonal (so with $0< \varepsilon < \delta$ small) we construct sub- and supermartingales for the stopped dynamics. The key statements are Propositions 3.14 and 3.16 below. The constructions rely on an analysis of $P_{(2)}$, which in turn is facilitated by the approximations (2.6) and (2.7) and a study of the corresponding linearized Koopman operator, defined for continuous real valued functions $\phi$ on ${\mathbb {T}\times \mathbb {R}^+}$ by

$$\begin{aligned} TP \phi (x,u) = \mathbb {E} \left[ \phi (T_\omega (x), DT_\omega (x)u)\right] . \end{aligned}$$

Formulas for the mentioned sub- and supermartingales are obtained from a study of the twisted Koopman operator ${P}_q$ whose action on a real valued function $\psi$ on $\mathbb {T}$ is defined as

$$\begin{aligned} {P}_q \psi (x) = \mathbb {E} \left[ \frac{\psi \left( T_\omega (x)\right) }{(D T_\omega (x))^q}\right] . \end{aligned}$$

This in turn connects to the moment Lyapunov exponent

$$\begin{aligned} \Lambda (q) = \lim _{n\rightarrow \infty } \frac{1}{n}\ln \left( \mathbb {E} \left[ D T_\omega ^n (x)^q \right] \right) . \end{aligned}$$

The moment Lyapunov exponent plays a central role in our analysis, similar to [11, 13]. Originally introduced by Molchanov [55] and subsequently developed in particular by Arnold [3], it can be viewed as a generalization of the Lyapunov exponent [25]. The relationship between the moment Lyapunov exponent and the Lyapunov exponent was first described in [26].

The above comments on methodology refer to a study of the two-point motion near the diagonal. Due to the non-injectivity of *T*, trajectories of distinct points $(x,y) \in \mathbb {T}^2 {\setminus } \Delta$ may land on the diagonal $\Delta$ in finite time. Our analysis circumvents this issue in two ways. First, the local estimates for the Koopman operators are restricted to strips $\Delta _{R} \setminus \Delta$ where $R < R_{min}$. Within this radius, the map acts injectively, ensuring that distinct points no further than *R* apart do not coalesce in a single iterate, allowing for well-defined expansion and contraction estimates. Second, regarding the global dynamics and measures construction in Sect. 5, the set of points that map directly onto $\Delta$ (and thus leave $\mathbb {T}^2 \setminus \Delta$) is controlled by Hypothesis (H4). This ensures that the probability of landing on or close to the diagonal from a large distance is negligible for our growth rate estimates.

## Koopman Operators

This section develops theory of Koopman operators needed for our analysis on random dynamics. The contents of this section are crucial for the arguments in the following sections, although the statements in the lemmas and propositions in the following sections can be read without reference to this section.

Below we define in particular the annealed Koopman operator and the linearized Koopman operator for the random one-point maps, and the annealed Koopman operator for the random two-point maps. What we refer to as the annealed Koopman operator is also known, especially in the stochastics literature, as the Markov semigroup. We consider the twisted Koopman operator and the moment Lyapunov exponent and use it to obtain eigenfunctions for the linearized Koopman operator. The approximation of the two-point random maps applied to nearby points by the linearized random map allows us to obtain, from these eigenfunctions, functions on which the two-point Koopman operator acts in a desired way: these functions appear in the construction of sub- and supermartingales for the random two-point maps considered near the diagonal.

### Twisted Koopman operator and moment Lyapunov function

Write $\mathcal {C}(\mathbb {T},\mathbb {R})$ for the space of real valued continuous functions on $\mathbb {T}$. Denote the $\mathcal C^0$-norm as $\Vert \cdot \Vert$. The *(annealed) Koopman operator* $P: \mathcal {C}(\mathbb {T},\mathbb {R})\rightarrow \mathcal {C}(\mathbb {T},\mathbb {R})$ is defined as

$$\begin{aligned} P \phi (x)&:=\mathbb {E} \left[ \phi (T_\omega (x) ) \right] . \end{aligned}$$

The *twisted Koopman operator*, ${P}_q: \mathcal {C}(\mathbb {T},\mathbb R) \rightarrow \mathcal {C}(\mathbb {T},\mathbb R)$ with $q \in \mathbb R$, is defined for continuous functions $\psi : \mathbb {T}\rightarrow \mathbb R$ by

$$\begin{aligned} {P}_q \psi (x) :=\mathbb {E} \left[ \frac{\psi \left( T_\omega (x)\right) }{(D T_\omega (x))^q}\right] . \end{aligned}$$

Note that $$P = {P}_0$$.

The following lemma establishes basic properties of $P_q$. Similar statements in other settings are in [3, 39].

#### Lemma 3.1

For $q \in \mathbb {R}$, the operator $P_q$ on $\mathcal {C}(\mathbb {T},\mathbb {R})$ is strictly positive and compact.

#### Proof

It is clear that $P_q \psi \ge 0$ if $\psi \ge 0$, that is, that $P_q$ is positive. Hypothesis (H2) yields the following property. There exists $n \in \mathbb {N}$, so that for any $\psi \in \mathcal {C}(\mathbb {T},\mathbb {R})$ with $\psi \ge 0$ and $\psi \ne 0$, $P_q^n \psi > 0$ everywhere.

This means that $P_q$ is strictly positive.

Let $\mathcal {B}$ be the unit ball in $\mathcal {C}(\mathbb {T},\mathbb {R})$. For $\psi \in \mathcal {B}$, note that (for readability skipping the $\pmod 1$ from the arguments)

$$\begin{aligned} P_q \psi (x) = \frac{1}{2\vartheta } \int _{-\vartheta }^\vartheta D T(x+\omega )^{-q} \psi (T(x+\omega ))\, d\omega = \frac{1}{2\vartheta } \int _{x-\vartheta }^{x+\vartheta } DT (y)^{-q} \psi (T(y))\, dy \end{aligned}$$

is a continuously differentiable function of $x \in \mathbb {T}$. As $\Vert P_q \psi \Vert \le C$ for a constant *C* (for $q>0$ we can take $C = a_2^{q}/\vartheta$, for $q<0$ we can take $C = a_1^{q}/\vartheta$ with $a_1,a_2$ from Hypothesis (H1)), $P_q$ is a bounded operator.

Now

$$\begin{aligned} \left| D P_q \psi (x) \right|&= \frac{1}{2\vartheta } \left| DT (x+\vartheta )^{-q} \psi (T(x+\vartheta )) - DT (x-\vartheta )^{-q} \psi (T(x-\vartheta )) \right| \\&\le C \Vert \psi \Vert \end{aligned}$$

for the same constant *C*. It follows that $P_q \psi$ for $\psi \in \mathcal {B}$ is an equicontinuous family of functions. By the Arzela–Ascoli theorem we get that $P_q$ is a compact operator.

Using this lemma we get the following results on the dominant eigenvalue and corresponding eigenvector of $P_q$.

We make use of the *q*th moment Lyapunov exponent $\Lambda (q)$ (see [3, 12, 39]) defined as

3.1 $$\begin{aligned} \Lambda (q) :=\lim _{n\rightarrow \infty } \frac{1}{n}\ln \left( \mathbb {E} \left[ D T_\omega ^n (x)^q \right] \right) . \end{aligned}$$

We also write moment Lyapunov function, especially when discussing its dependence on *q*. We will find that the limit does not depend on *x*, and that it exists as an analytic function of *q*. Denote $1\!\!1_\mathbb {T}:\mathbb {T}\rightarrow \mathbb {R}$ as the constant function equal to 1 on the circle.

#### Proposition 3.2

For $q \in \mathbb {R}$, ${P}_{q}$ has a dominant simple eigenvalue $e^{\Lambda (-q)}$. The rest of the spectrum of $P_q$ is contained in a disk of radius less then $e^{\Lambda (-q)}$. Write $\phi _q \in \mathcal {C} (\mathbb {T},\mathbb R)$ for the dominant eigenfunction of ${P}_{q}$, so

3.2 $$\begin{aligned} {P}_{q}\phi _{q} = e^{\Lambda (-q)} \phi _{q}. \end{aligned}$$

Then

1. $$\phi _0 = 1\!\!1_\mathbb {T}$$ and $$e^{\Lambda (0)} = 1$$,
2. $\phi _q$ is a positive function,
3. $\Lambda (q)$ and $\phi _{q}$ depend analytically on *q*,
4. $$\Lambda $$ is convex,
5. $$\Lambda (q) \ge \lambda q$$.

#### Proof

The proof follows ideas as in [3, 39]. The spectral properties of $P_q$ follow from Lemma 3.1 and the Krein-Rutman theorem (see for instance [30, Section 19.5]). Write $r(q) = \sigma \left( P_{q} \right)$ for the spectral radius of $P_q$. By the Krein-Rutman theorem, this equals the dominant eigenvalue of $P_q$. The dominant eigenvalue is simple, because the operator is strictly positive. We have

$$\begin{aligned} P^n_q1\!\!1_\mathbb {T} (x) = \langle k_q, 1\!\!1_\mathbb {T} \rangle r(q)^n \phi _q (x) + o(r(q)^n), \end{aligned}$$

as $n\rightarrow \infty$, uniformly in *x*, where $k_q\in \mathcal C(\mathbb {T},\mathbb {R})^*$ is a probability measure, see [3]. Now we rescale $\phi _q$, such that $\langle k_q, 1\!\!1_\mathbb {T} \rangle =1$ and using this, calculate

$$\begin{aligned} \lim _{n\rightarrow \infty } \frac{1}{n} \ln \left( \mathbb {E} \left[ \left( DT_\omega ^n (x) \right) ^{-q} \right] \right)&= \lim _{n\rightarrow \infty }\frac{1}{n} \ln \left( P^n_q1\!\!1_\mathbb {T} (x) \right) \\&= \lim _{n\rightarrow \infty }\ln \left( \left( P^n_q1\!\!1_\mathbb {T} (x) \right) ^{1/n} \right) \\&= \lim _{n\rightarrow \infty } \ln \left( \left( r(q)^n \phi _q (x) + o(r(q)^n) \right) ^{1/n} \right) \\&= \ln (r(q)). \end{aligned}$$

We find that $\lim _{n\rightarrow \infty } \frac{1}{n} \ln \left( \mathbb {E} \left[ \left( DT_\omega ^n (x) \right) ^{-q} \right] \right)$ does not depend on *x*. The remaining properties of $\Lambda (-q)$ and $\phi _q$ follow from [3, 39].

The following lemma connects the moment Lyapunov exponent and the Lyapunov exponent.

#### Lemma 3.3

The first derivative of the moment Lyapunov function at $q=0$ is equal to the Lyapunov exponent

3.3 $$\begin{aligned} \Lambda ' (0)&= \lim _{n\rightarrow \infty } \frac{1}{n} \mathbb {E} \left[ \ln \left( D T_\omega ^n (x) \right) \right] = \lambda . \end{aligned}$$

#### Proof

This follows from the fact that $\Lambda (0) =0$, $\Lambda (q) \ge \lambda q$ and the analyticity of $\Lambda$. For the complete argument, see [3, 39].

We have established that $\Lambda$ is a convex function that vanishes at 0. The following lemma shows the existence of a second zero if $\lambda \ne 0$. This second zero plays a prominent role in our analysis and also appears in the statements of the main theorem on stationary measures.

#### Lemma 3.4

If $\lambda \ne 0$ then there is a unique $\gamma \ne 0$, with opposite sign, such that $\Lambda (\gamma ) = 0$. (We set $\gamma = 0$ if $\lambda = 0$.)

#### Proof

Assume that $\lambda > 0$, which implies by (3.3) that $\Lambda '(0) > 0$. By Hypothesis (H3) there is a contracting periodic point $x_c$ of *T*. Let $k_c$ denote its period. By continuity of the map, there exist $\varepsilon > 0$ and $\delta > 0$ such that the set

$$\begin{aligned} \mathcal {H}^c = \big \{ \omega \in \Sigma _\vartheta \;; \; x \in B_\varepsilon (x_c) \text { implies } T^{k_c}_\omega (x) \in B_\varepsilon (x_c),\, \ln (DT_\omega ^{k_c}(x)) < -\delta \big \} \end{aligned}$$

has positive measure $\mathbb {P} ( \mathcal {H}^c ) > 0$.

By Hypothesis (H2), we know that for any $x \in \mathbb {T}$, there is a positive probability of reaching $B_\varepsilon (x_c)$ in *k* steps. For any $m = k + nk_c$ sufficiently large, we have

$$\begin{aligned} \mathbb {E} \left[ (DT_\omega ^m(x))^{-q} \right]&\ge \mathbb {P}(x_k \in B_\varepsilon (x_c))\, e^{-n \delta q}\, \mathbb {P}(\mathcal {H}^c)^n. \end{aligned}$$

Taking the logarithm and applying the limit $\lim _{n \rightarrow \infty } \frac{\ln (\cdot )}{n}$, it follows that $\Lambda (q) \rightarrow \infty$ as $q \rightarrow -\infty$. By continuity of $\Lambda (q)$ and the fact that $\Lambda '(0) > 0$, there is a unique $\gamma < 0$ such that $\Lambda (\gamma ) = 0$.

The argument in the case where $\lambda < 0$ is analogous, using an expanding periodic orbit instead of the contracting periodic orbit. The existence of an expanding periodic orbit $x_c$ of *T* follows from [54]. Let $k_c$ denote its period. For $\omega _0,\ldots ,\omega _{k_c-1}$ near 0, the maps $T^{k_c}_\omega$ have hyperbolic repelling fixed points near $x_c$. Since $\frac{\partial }{\partial \omega } T_\omega > 0$ everywhere, these hyperbolic repelling fixed points form a set that contains an open neighborhood of $x_c$. Using this, there exist $\varepsilon >0$, $\delta >0$ and $C>0$, such that for each $x \in B_\varepsilon (x_c)$ the set

$$\begin{aligned} \mathcal {H}^c_{x} = \big \{ \omega \in \Sigma _\vartheta \;; \; T^{k_c}_\omega (x) \in B_\varepsilon (x_c),\, \ln (DT_\omega ^{k_c}(x)) > \delta \big \} \end{aligned}$$

has strictly positive measure $\mathbb {P} (\mathcal {H}^c_{x}) > C$. The proof can now be finished as above. $\square$

We write $\partial _q \phi _q$ for the derivative of $\phi _q$ with respect to *q*, and likewise $\partial ^2_q \phi _q$ for the second order derivative with respect to *q*. Recall from (3.3) that $\lambda = \Lambda '(0)$ and let

#### Lemma 3.5

We have the following two equalities for $x \in \mathbb {T}$:

3.4 $$\begin{aligned} \mathbb {E}\left[ \ln (D T_\omega (x) ) \right] -\lambda = \left( P_0 - I\right) {\partial _q \phi _0} (x) \end{aligned}$$

and, if $$\lambda = 0$$,

3.5 $$\begin{aligned} V = \left( P_0 - I\right) {\partial _q^2 \phi _0} (x)- 2 \mathbb {E}\left[ {\partial _q \phi _0} (T_\omega (x)) \ln (D T_\omega (x)) \right] + \mathbb {E}\left[ \ln ^2(DT_\omega (x))\right] . \end{aligned}$$

#### Proof

Differentiating (3.2) once with respect to *q*, yields for $x \in \mathbb {T}$,

3.6 $$\begin{aligned} e^{\Lambda (-q)}{\partial _q \phi _q} (x) - e^{\Lambda (-q)}\Lambda '(-q)\phi _q(x)&= \mathbb {E}\left[ \partial _q\left( \frac{\phi _q\left( T_\omega (x)\right) }{D T_\omega (x) ^q}\right) \right] \nonumber \\&= ({P}_{q}{\partial _q \phi _q} )(x) - \mathbb {E}\left[ \frac{\phi _q\left( T_\omega (x)\right) \ln (DT_\omega (x))}{D T_\omega (x)^q}\right] . \end{aligned}$$

Note that the derivative of $\phi _q(x),$ with respect to *q*, exists because of Proposition 3.2.

Equation (3.6) evaluated at $q = 0$ yields

$$\begin{aligned} {\partial _q \phi _0} (x) - \Lambda '(0) = ({P}_{0}{\partial _q \phi _0} )(x) - \mathbb {E} \left[ \ln (DT_\omega (x)) \right] . \end{aligned}$$

Rewriting this proves (3.4).

Next, differentiating (3.6) with respect to *q* yields

3.7 $$\begin{aligned}  &   e^{\Lambda (-q)}\left( {\partial ^2_q \phi _q} - 2\Lambda '(-q) {\partial _q \phi _q} + \left( \Lambda '(-q)^2 +\Lambda ''(-q) \right) \phi _q \right) (x) \nonumber \\  &   \quad = {P}_q{\partial _q^2 \phi _q} (x) - 2\mathbb {E} \left[ \frac{{\partial _q \phi _q} \left( T_\omega (x) \right) \ln (DT_\omega (x))}{D T_\omega (x)^q}\right] + \mathbb {E}\left[ \frac{\phi _q\left( T_\omega (x) \right) \ln ^2(D T_\omega (x))}{D T_\omega (x)^q}\right] .\nonumber \\ \end{aligned}$$

Evaluating (3.7) for $q=0$ yields

3.8 $$\begin{aligned}  &   - 2\Lambda '(0) {\partial _q \phi _0} (x) + \Lambda '(0)^2 + \Lambda ''(0) \nonumber \\  &   \quad = \left( {P}_0 - I \right) {\partial _q^2 \phi _0} (x) - 2\mathbb {E} \left[ {\partial _q \phi _0} \left( T_\omega (x) \right) \ln (DT_\omega (x)) \right] + \mathbb {E}\left[ \ln ^2(D T_\omega (x))\right] .\qquad \quad \end{aligned}$$

Rewriting, and plugging in $\lambda =0$, we obtain (3.5). $\square$

It is immediate from the convexity of $\Lambda$ that $V \ge 0$. Our set-up implies that in fact $V>0$, which we require in our analysis of the case with a zero Lyapunov exponent $\lambda$ (see Lemma 4.1 below).

#### Lemma 3.6

We have $$V>0$$.

#### Proof

Following [12] we establish that $V = 0$ implies $\Lambda (q) = \lambda q$, which will lead to a contradiction.

Rewriting (3.8), we obtain

3.9 $$\begin{aligned}  &   V + \left( P_0 - I\right) \left( (\partial _q \phi _0 )^2 - \partial ^2_q \phi _0 \right) (x) \nonumber \\  &   \quad = \mathbb {E}\left[ \left( \partial _q \phi _0 (T_\omega (x)) - \ln (DT_\omega (x)) \right) ^2 \right] - \left( \partial _q \phi _0 (x) - \lambda \right) ^2 \nonumber \\  &   \quad = \mathbb {E}\left[ \left( \partial _q \phi _0 (T_\omega (x)) - \ln (DT_\omega (x)) \right) ^2 \right] - \left( \mathbb {E}\left[ \partial _q \phi _0 (T_\omega (x)) - \ln (DT_\omega (x)) \right] \right) ^2.\nonumber \\ \end{aligned}$$

The last step uses (3.4). We conclude that

$$\begin{aligned} V + \left( P_0 - I\right) \left( (\partial _q \phi _0 )^2 - \partial ^2_q \phi _0 \right) (x) \ge 0. \end{aligned}$$

Now suppose $V=0$, for the sake of contradiction. From $\left( P_0 - I\right) \left( (\partial _q \phi _0 )^2 - \partial ^2_q \phi _0 \right) (x) \ge 0$ we conclude that $(\partial _q \phi _0 )^2 - \partial ^2_q \phi _0$ is constant (to see this, note that if a continuous function $\chi :\mathbb {T} \rightarrow \mathbb {R}$ takes a maximum at *x*, then the property $\mathbb {E} \left[ \chi (T_\omega (x)) \right] \ge \chi (x)$ with Hypothesis (H2) implies that $\chi$ is maximal at every point of $\mathbb {T}$). So in fact

$$\begin{aligned} \left( P_0 - I\right) \left( (\partial _q \phi _0 )^2 - \partial ^2_q \phi _0 \right) (x) = 0 \end{aligned}$$

and (3.9) implies that

$$\begin{aligned} \partial _q \phi _0 (T_\omega (x)) - \ln (DT_\omega (x)) = \partial _q \phi _0 (x) - \lambda \end{aligned}$$

for all *x* and $\omega$. Then also

$$\begin{aligned} \partial _q \phi _0 (T^n_\omega (x)) - \ln (DT^n_\omega (x)) = \partial _q \phi _0 (x) - n\lambda . \end{aligned}$$

As $\Lambda (q) = \lim _{n\rightarrow \infty } \frac{1}{n} \ln \left( \mathbb {E} \left[ \left( DT^n_\omega (x) \right) ^q \right] \right)$ we find $\Lambda (q) = q \lambda$. Lemma 3.4 shows that this is not the case in our set-up. $\square$

### Linearized Koopman operator

We introduce the *linearized Koopman operator*, defined for continuous real valued functions $\phi$ on ${\mathbb {T}\times \mathbb {R}^+}$ by

$$\begin{aligned} TP \phi (x,u) :=\mathbb {E} \left[ \phi (T_\omega (x), DT_\omega (x)u)\right] . \end{aligned}$$

Recall from Proposition 3.2 that $\phi _q$ is the dominant eigenfunction of ${P}_q$. Define $\tilde{W}_q:{\mathbb {T}\times \mathbb {R}^+}\rightarrow \mathbb {R}$ by

3.10 $$\begin{aligned} \tilde{W}_q(x,u)&= u^{-q}\phi _q\left( x\right) . \end{aligned}$$

We reserve the *tilde* notation for functions on $\mathbb {T}\times \mathbb {R}^+$.

#### Lemma 3.7

The function $\tilde{W}_q$ is an eigenfunction of *TP*, with eigenvalue ${e^{\Lambda (-q)}}$:

3.11 $$\begin{aligned} TP \tilde{W}_q (x,u) = e^{\Lambda (-q)} \tilde{W}_q (x,u). \end{aligned}$$

#### Proof

This follows from a straightforward computation. For $(x,u) \in {\mathbb {T}\times \mathbb {R}^+}$,

$$\begin{aligned} TP \tilde{W}_q (x,u)&= \mathbb {E} \left[ \tilde{W}_q(T_\omega (x),D T_\omega (x) u)\right] \\&= \mathbb {E} \left[ (DT_\omega (x) u)^{-q}\phi _q\left( T_\omega (x)\right) \right] \\&= u^{-q}\mathbb {E} \left[ DT_\omega (x) ^{-q}\phi _q\left( T_\omega (x)\right) \right] \\&= u^{-q} {P}_q\phi _q(x)\\&= e^{\Lambda (-q)} u^{-q} \phi _q(x)\\&= e^{\Lambda (-q)}\tilde{W}_q(x,u). \end{aligned}$$

#### Remark 3.8

Consider a random sequence

$$\begin{aligned} (x_{n+1}, u_{n+1}) = \left( T_{\sigma ^n\omega } (x_n), DT_{\sigma ^n\omega } (x_n) u_n\right) , \end{aligned}$$

with $x_0,u_0 \in \mathbb {T}\times \mathbb {R}^+$. Take $\gamma$ as in Lemma 3.4 such that $e^{\Lambda (\gamma )}=1$. Then

$$\begin{aligned} (TP - I) \tilde{W}_\gamma (x,u) = 0, \end{aligned}$$

which shows that $\tilde{W}_\gamma (x_n,u_n)$ is a martingale. $\square$

#### Example 3.9

In the setting of Example 1.3 with $\vartheta = 0.5$, we obtain

$$\begin{aligned} \tilde{W}_q(x,u) = \Vert \phi _q\Vert \,u^{-q}, \end{aligned}$$

which is independent of *x*, since $P_q\psi$ is constant for every $\psi \in \mathcal C(\mathbb {T},\mathbb {R})$. $\square$

#### Lemma 3.10

There exist $K>0$ and two continuous functions

$$\begin{aligned} \tilde{\phi }, \tilde{\eta } \in \mathcal {C}^0 ({\mathbb {T}\times \mathbb {R}^+},\mathbb R), \end{aligned}$$

such that the following holds for $(x,u) \in {\mathbb {T}\times \mathbb {R}^+}$. For $\tilde{\phi }$ we have

3.12 $$\begin{aligned} \vert \tilde{\phi }(x,u) - \ln (u) \vert&\le K, \end{aligned}$$

3.13 $$\begin{aligned} \left( TP-I\right) \tilde{\phi } (x,u)&=\lambda . \end{aligned}$$

Assume $\lambda = 0$. Then for $\tilde{\eta }$ we have

3.14 $$\begin{aligned} \vert \tilde{\eta }(x,u) - \ln ^2(u) \vert&\le K|\ln (u)|, \end{aligned}$$

3.15 $$\begin{aligned} \left( TP-I\right) \tilde{\eta }(x,u)&= V. \end{aligned}$$

Concerning notation in the following proof and further below, $\pi _1$ is the function $\pi _1 (x,u) = x$ on $\mathbb {T}\times \mathbb {R}^+$, $\pi _2$ stands for the function $\pi _2 (x,u) = u$ on $\mathbb {T}\times \mathbb {R}^+$.

#### Proof of Lemma 3.10

We give a constructive proof for Lemma 3.10, where we use Proposition 3.2 and Lemma 3.5. We first find the function $\tilde{\phi }$ for which (3.12) and (3.13) holds. Subsequently we find $\tilde{\eta }$ for which (3.14) and (3.15) holds.

For $$ (x,u) \in {\mathbb {T}\times \mathbb {R}^+}$$, let

3.16 $$\begin{aligned} \tilde{\phi }(x,u)&= \ln (u) -{\partial _q \phi _0} (x). \end{aligned}$$

Note that

$$\begin{aligned} \tilde{\phi }\in \mathcal {C}^0 ({\mathbb {T}\times \mathbb {R}^+},\mathbb R). \end{aligned}$$

Furthermore, for all $(x,u) \in {\mathbb {T}\times \mathbb {R}^+}$, we have

$$\begin{aligned} \left( TP-I\right) \tilde{\phi } (x,u)&= \left( TP-I\right) \ln (\pi _2)(x,u) -\left( P_0-I\right) \left( {\partial _q \phi _0} \right) (x) \\&= \mathbb {E} \left[ \ln ( DT_\omega (x) u )\right] - \ln (u) - \mathbb {E} \left[ \ln ( DT_\omega (x) )\right] + \lambda \\&=\lambda . \end{aligned}$$

In the first line we use that ${\partial _q \phi _0} (x)$ only depends on *x*. For the second equality we apply (3.4) in Lemma 3.5.

For the second part of the proof, we take $\lambda =0$ and let

$$\begin{aligned} \tilde{\eta }(x,u) = \ln ^2(u) -2\ln (u){\partial _q \phi _0} (x) +{\partial ^2_q \phi _0} (x). \end{aligned}$$

And again note that

$$\begin{aligned} \tilde{\eta }\in \mathcal {C}^0 ({\mathbb {T}\times \mathbb {R}^+},\mathbb R). \end{aligned}$$

From a straightforward computation, we obtain, for $(x,u)\in {\mathbb {T}\times \mathbb {R}^+}$

$$\begin{aligned}  &   \left( TP-I\right) \eta (x,u) = \left( TP-I\right) \ln ^2(\pi _2)(x,u)\\  &   \quad - 2 \left( TP-I\right) \ln (\pi _2){\partial _q \phi _0} (\pi _1)(x,u) + \left( TP-I\right) {\partial ^2_q \phi _0} (\pi _1)(x,u). \end{aligned}$$

We analyse the terms on the right hand side separately. For the first term, we have

3.17 $$\begin{aligned} \left( TP-I\right) \left( \ln ^2(\pi _2)\right) (x,u)&= \mathbb {E} \left[ \ln ^2(DT_\omega (x) u)\right] - \ln ^2(u) \nonumber \\&= \mathbb {E} \left[ \left( \ln (DT_\omega (x)) + \ln (u)\right) ^2\right] - \ln ^2(u) \nonumber \\&= \mathbb {E} \left[ \ln ^2(DT_\omega (x)) \right] + 2 \ln (u) \mathbb {E} \left[ \ln (DT_\omega (x)) \right] . \end{aligned}$$

For the second term we have

3.18 $$\begin{aligned} 2\left( TP-I\right)&\ln (\pi _2){\partial _q \phi _0} (\pi _1)(x,u) = 2\mathbb {E} \left[ \ln (DT_\omega (x)u){\partial _q \phi _0} (T_\omega (x)) \right] - 2\ln (u){\partial _q \phi _0} (x) \nonumber \\&= 2\mathbb {E} \left[ \ln (DT_\omega (x)){\partial _q \phi _0} (T_\omega (x)) \right] +2 \ln (u)\left( \mathbb {E} \left[ {\partial _q \phi _0} (T_\omega (x))\right] - {\partial _q \phi _0} (x) \right) \nonumber \\&= 2\mathbb {E} \left[ \ln (DT_\omega (x)){\partial _q \phi _0} (T_\omega (x)) \right] +2 \ln (u) (P-I) {\partial _q \phi _0} (x) \nonumber \\&= 2\mathbb {E} \left[ \ln (DT_\omega (x)){\partial _q \phi _0} (T_\omega (x)) \right] + 2\ln (u)\mathbb {E} \left[ \ln (DT_\omega (x)) \right] , \end{aligned}$$

where in the third to fourth line we make use of the properties of ${\partial _q \phi _0}$, as described by (3.4) in Lemma 3.5. For the third term, we have

3.19 $$\begin{aligned} \left( TP-I\right) {\partial ^2_q \phi _0} (\pi _1)(x,u)&=V +2\mathbb {E}\left[ \ln (DT_\omega (x) ){\partial _q \phi _0} \left( T_\omega (x)\right) \right] - \mathbb {E} \left[ \ln ^2(DT_\omega (x) )\right] . \end{aligned}$$

Here we use (3.5) in Lemma 3.5. Combining (3.17), (3.18) and (3.19), we conclude, for $(x,u)\in {\mathbb {T}\times \mathbb {R}^+}$,

$$\begin{aligned} \left( TP-I\right) \tilde{\eta }(x,u) = V . \end{aligned}$$

Finally, (3.12) and (3.14) follow from the analyticity in *q* of $\phi _q(x)$(see Proposition 3.2).

#### Remark 3.11

Consider a random sequence

$$\begin{aligned} (x_{n+1}, u_{n+1}) = \left( T_{\sigma ^n\omega } (x_n), DT_{\sigma ^n\omega } (x_n) u_n\right) , \end{aligned}$$

with $(x_0,u_0) \in \mathbb {T}\times \mathbb {R}^+$. Then (3.13) expresses that the function $\tilde{\phi }(x_n,u_n) - n\lambda$ is a martingale. Assuming $\lambda =0$, (3.15) expresses that the function $\tilde{\eta }(x_n,u_n) - nV$ is a martingale. $\square$

#### Example 3.12

In the case of Example 1.3, with $\vartheta = 0.5$, we get $\tilde{\phi } (x,u) = \ln (u) + \Vert \partial _q\phi _0\Vert$ and $\tilde{\eta }(x,u) = \ln ^2(u)-2\Vert \partial _q\phi _0\Vert \ln (u) + \Vert \partial _q^2\phi _0\Vert$. Both functions are independent of *x*, as $P_q\psi$ is constant for every $\psi \in \mathcal C(\mathbb {T},\mathbb {R})$. $\square$

### Two-point Koopman operator

The *(annealed) two-point Koopman operator* $P_{(2)}$ is defined for a real valued function $\phi$ on $\mathbb {T}^2\setminus \Delta$ by

$$\begin{aligned} P_{(2)}\phi (x,y) :=\mathbb {E} \left[ \phi (T_\omega ^{(2)}(x,y))\right] . \end{aligned}$$

Define $$\phi :\mathbb {T}^2 \setminus \Delta \rightarrow \mathbb {R}$$ by

$$\begin{aligned} \phi (x,y) :=\ln (d(x,y)) - {\partial _q \phi _0} (x). \end{aligned}$$

With $\tilde{\phi }$ from Lemma 3.10 (see (3.16)) we have, for $(x,y) \in \mathbb {T}^2 \setminus \Delta$,

3.20 $$\begin{aligned} \phi (x,y) = \tilde{\phi }(x,d(x,y)). \end{aligned}$$

Let $\eta :\mathbb {T}^2 \setminus \Delta \rightarrow \mathbb {R}$ be defined as

$$\begin{aligned} \eta (x,y) :=\ln ^2(d(x,y)) -2{\partial _q \phi _0} (x)\ln (d(x,y)) + {\partial _q \phi _0} (x). \end{aligned}$$

Note that for $(x,y) \in \mathbb {T}^2 \setminus \Delta$,

3.21 $$\begin{aligned} \eta (x,y) = \tilde{\eta }(x,d(x,y)). \end{aligned}$$

Define $$W_q: \mathbb {T}^2\setminus \Delta \rightarrow \mathbb {R}$$ by

3.22 $$\begin{aligned} W_q(x,y) :=d(x,y)^{-q}\phi _q(x). \end{aligned}$$

With $\tilde{W}_q$ from (3.10) we have for $(x,y) \in \mathbb {T}^2{\setminus }\Delta$,

3.23 $$\begin{aligned} W_q(x,y) = \tilde{W}_q(x,d(x,y)). \end{aligned}$$

The next lemma compares the action of *TP* and $P_{(2)}$.

#### Lemma 3.13

There exist $R,B>0$, such that we have the following bounds, for $(x,y)\in \Delta _R\setminus \Delta$,

3.24 $$\begin{aligned} \left| TP\tilde{\phi }(x,d(x,y)) - P_{(2)}\phi (x,y)) \right|&\le B d(x,y), \end{aligned}$$

3.25 $$\begin{aligned} \left| TP\tilde{\eta }(x,d(x,y)) - P_{(2)} \eta (x,y)\right|&\le B d(x,y)|\ln (d(x,y))|, \end{aligned}$$

and for $$q \in [-|\gamma | -1, |\gamma | +1 ]$$,

3.26 $$\begin{aligned} \left| TP\tilde{W}_q(x,d(x,y))-P_{(2)}W_q(x,y)\right| \le B d(x,y)^{-q+1}. \end{aligned}$$

#### Proof

We will work out the estimates for (3.24), then the computation for (3.25) is analogous. We will sketch the proof for (3.26).

Recall that *w*(*x*, *y*) denotes the signed distance between *x* and *y*, for nearby points $x, y \in \mathbb {T}$. Take $R >0$ small enough so that $T_\omega$ is injective on all intervals of length *R*. For $(x,y) \in \Delta _{R}\setminus \Delta$,

3.27 $$\begin{aligned} TP \tilde{\phi }(x,|w(x,y)|)&= \mathbb {E} \left[ \ln (|DT_\omega (x) w(x,y)| )\right] - P_0{\partial _q \phi _0} (x) \nonumber \\&= \ln (|w(x,y)|) + \mathbb {E} \left[ \ln (DT_\omega (x)) \right] -\mathbb {E} \left[ {\partial _q \phi _0} \left( T_\omega (x)\right) \right] . \end{aligned}$$

To determine $P_{(2)}\phi$, we use a Taylor expansion for $T_\omega$. We obtain,

$$\begin{aligned} w(T_\omega ^{(2)}(x,y)) = DT_\omega (x)w(x,y) +\frac{1}{2} D^2 T_\omega (\xi )w(x,y)^2 , \end{aligned}$$

for a $\xi$ in the interval between *x* and $x + w(x,y)$. Therefore, for $(x,y) \in \Delta _{R}\setminus \Delta$ with $R>0$ small enough,

3.28 $$\begin{aligned}  &   P_{(2)} \phi (x,y) = \mathbb {E} \left[ \ln \left( \left| w(T_\omega ^{(2)}(x,y))\right| \right) \right] -P_0{\partial _q \phi _0} (x) \nonumber \\  &   \quad = \mathbb {E} \left[ \ln \left( \left| DT_\omega (x) w(x,y) + \frac{D^2T_\omega (\xi ) w(x,y)^2}{2} \right| \right) \right] - P_0{\partial _q \phi _0} (x) \nonumber \\  &   \quad = \ln (|w(x,y)|) + \mathbb {E} \left[ \ln \left( DT_\omega (x)\right) \right] + \mathbb {E} \left[ \ln \left( \left| 1 +\frac{D^2T_\omega (\xi ) w(x,y)}{2 DT_\omega (x)} \right| \right) \right] \nonumber \\  &   \qquad - \mathbb {E} [{\partial _q \phi _0} (T_\omega (x))]. \end{aligned}$$

Combining (3.27) and (3.28) yields, for $(x,y) \in \Delta _{R}\setminus \Delta$,

3.29 $$\begin{aligned} \left| P_{(2)} \phi (x,y) - TP \phi (x,|w(x,y)|) \right|&\le \left| \mathbb {E} \left[ \ln \left( \left| 1 +\frac{D^2T_\omega (\xi ) w(x,y)}{2 DT_\omega (x)} \right| \right) \right] \right| . \end{aligned}$$

The right hand side of (3.29) can be bounded using the standard inequalities $x/(1+x)\le \ln (1+x) \le x$ for $x>-1$. For *R* small we have $\left| \frac{D^2 T_\omega (\xi ) w(x,y)}{2DT_\omega (x)}\right| < 1$ for $(x,y) \in \Delta _{R}{\setminus } \Delta$. Then for the upper bound we obtain

$$\begin{aligned} \mathbb {E} \left[ \ln \left( 1 + \frac{D^2 T_\omega (\xi ) w(x,y)}{2DT_\omega (x)}\right) \right]&\le \frac{|w(x,y)| \Vert D^2T_\omega \Vert }{2 a_1}. \end{aligned}$$

Similarly for the lower bound, when we take *R* small enough so that $|w(x,y)| \Vert D^2 T_\omega \Vert < a_1$ for $(x,y) \in \Delta _{R}{\setminus } \Delta$, we obtain

$$\begin{aligned} \mathbb {E} \left[ \ln \left( 1 + \frac{D^2T_\omega (\xi ) w(x,y)}{2DT_\omega (x)} \right) \right]&\ge \mathbb {E} \left[ \frac{\displaystyle \frac{D^2T_\omega (\xi ) w(x,y)}{2DT_\omega (x)}}{\displaystyle 1 + \frac{D^2T_\omega (\xi ) w(x,y)}{2DT_\omega (x)}} \right] \\&\ge \mathbb {E} \left[ \dfrac{D^2T_\omega (\xi ) w(x,y)}{2DT_\omega (x) + D^2T_\omega (\xi ) w(x,y)}\right] \\&\ge -|w(x,y)|\Vert D^2T_\omega \Vert \mathbb {E} \left[ \frac{1}{2DT_\omega (x) + D^2T_\omega (\xi ) w(x,y)}\right] \\&\ge \frac{-|w(x,y)|\Vert D^2T_\omega \Vert }{a_1}. \end{aligned}$$

Setting $B = \frac{\Vert D^2T_\omega \Vert }{a_1}$ finishes the estimates for (3.24).

To prove (3.25), we again perform an explicit calculation. Note that $\eta = \ln ^2(d(x,y)) - 2 \partial _q \phi _0 (x)\ln (d(x,y)) + \partial _q\phi _0(x)$ is made up from three terms. When bounding the difference $\left| TP\tilde{\eta }(x,d(x,y)) - P_{(2)} \eta (x,y)\right|$, the contribution from the last two terms is treated as above.

It therefore suffices to bound the contribution from the first term $\ln ^2(d(x,y))$.

For $(x,y) \in \Delta _{R}\setminus \Delta$, factoring the Taylor expansion as in the previous step, we have

$$\begin{aligned} P_{(2)} \ln ^2(d) (x,y)&= \mathbb {E} \left[ \ln ^2\left( \left| DT_\omega (x)w(x,y) + \frac{D^2T_\omega (\xi ) w(x,y)^2}{2} \right| \right) \right] \\&= \mathbb {E} \left[ \left( \ln (|DT_\omega (x)w(x,y)|) + \ln \left( \left| 1 +\frac{D^2T_\omega (\xi ) w(x,y)}{2 DT_\omega (x)} \right| \right) \right) ^2 \right] . \end{aligned}$$

Expanding the square and comparing with the term $\mathbb {E}[\ln ^2(|DT_\omega (x)w(x,y)|)]$ from $TP \tilde{\eta }$, we find that the difference is bounded by

$$\begin{aligned}&\left| P_{(2)} \eta (x,y) - TP \tilde{\eta } (x,d(x,y)) \right| \\&\quad \le \mathbb {E} \left[ \left| 2\ln (|DT_\omega (x)w(x,y)|) \ln \left( 1 +\frac{D^2T_\omega (\xi ) w(x,y)}{2 DT_\omega (x)} \right) \right| \right] \\&\qquad + \mathbb {E} \left[ \ln ^2\left( 1 +\frac{D^2T_\omega (\xi ) w(x,y)}{2 DT_\omega (x)} \right) \right] + O(d(x,y)). \end{aligned}$$

Using the inequality $u^2/(1+u)^2\le \ln ^2(1+u) \le u^2$ for small *u* (as in (3.29)), the second term is bounded by $O(d(x,y)^2)$. For the first term, since *R* is small and $|\ln (|DT_\omega (x)|)|$ is bounded, we have $|\ln (|DT_\omega (x)w(x,y)|)| \le C |\ln (d(x,y))|$ for some constant *C*. Thus,

$$\begin{aligned} \left| P_{(2)} \eta (x,y) - TP \tilde{\eta } (x,d(x,y)) \right|&\le 2 C |\ln (d(x,y))| \cdot \frac{|w(x,y)| \Vert D^2T_\omega \Vert }{2 a_1} + O(d(x,y)) \\&\le B d(x,y) |\ln (d(x,y))|. \end{aligned}$$

We can choose *B* such that (3.25) holds for $(x,y) \in \Delta _{R}\setminus \Delta$ with *R* small enough.

For (3.26), take $(x,y) \in \Delta _{R}\setminus \Delta$ with *R* small and assume, without loss of generality, $x<y$. Then

$$\begin{aligned}  &   \left| TP\tilde{W_q}(x,d(x,y))-P_{(2)}W_q(x,y)\right| \nonumber \\  &   \quad \le \left\| \phi _q\right\| \mathbb {E} \left[ \left| \left| DT_\omega (x) w(x,y) \right| ^{-q} - \left| DT_\omega (x) w(x,y) +\frac{1}{2} D^2T_\omega (\xi ) w(x,y)^2 \right| ^{-q} \right| \right] . \nonumber \end{aligned}$$

With () and the mean value theorem for $a \mapsto a^{-q}$, we get

$$\begin{aligned} \left| TP\tilde{W_q}(x,w(x,y))-P_{(2)}W_q(x,y)\right|&\le C_q |q| \mathbb {E} \left[ |w(x,y)|^{-q-1}|D^2T_\omega (\xi )w(x,y)|^2 \right] \\&\le C_q |q| \Vert D^2T \Vert ^2 |w(x,y)|^{-q+1} \\&\le B d(x,y)^{-q+1}, \end{aligned}$$

for some positive constant $C_q$. As $C_q|q|$ depends continuous on *q*, restricting *q* to $[-|\gamma |-1,|\gamma |+1]$ allows us to uniformly bound it with a constant *B*. This completes the proof. $\square$

The following lemma is a key lemma that adapts the equalities and estimates in Lemma 3.13 for *TP* to the setting for $P_{(2)}$, but only near the diagonal $\Delta$, using that near $\Delta$, $P_{(2)}$ can be approximated by *TP* (Lemma 3.13).

#### Proposition 3.14

There exists a $R,K>0$, and continuous integrable functions

$$\begin{aligned} \phi ^\pm , \eta ^\pm \in \mathcal {C}^0(\mathbb {T}^2\setminus \Delta , \mathbb {R}), \end{aligned}$$

such that the following holds for $(x,y) \in \Delta _R\setminus \Delta$.

For $\phi ^\pm$ we have

3.30 $$\begin{aligned} \vert \phi ^\pm (x,y) - \ln (d(x,y)) \vert&\le K, \end{aligned}$$

3.31 $$\begin{aligned} \left( P_{(2)}-I\right) \phi ^- (x,y)&\le \lambda \le \left( P_{(2)}-I\right) \phi ^+ (x,y). \end{aligned}$$

Assume $\lambda =0$. Then for $\eta ^\pm$ we have

3.32 $$\begin{aligned} \vert \eta ^\pm (x,y) - \ln ^2(d(x,y)) \vert&\le K|\ln (d(x,y))| , \end{aligned}$$

3.33 $$\begin{aligned} \left( P_{(2)}-I\right) \eta ^- (x,y)&\le V \le \left( P_{(2)}-I\right) \eta ^+ (x,y). \end{aligned}$$

#### Proof

We can bound $\left( P_{(2)}-I\right) \phi (x,y)$, by applying the triangle inequality and using (3.13), (3.20) and (3.24):

$$\begin{aligned}  &   \left| \left( P_{(2)}-I\right) \phi (x,y) - \lambda \right| \le \overbrace{\left| TP\tilde{\phi }(x,d(x,y))-P_{(2)}\phi (x,y)\right| }^{\le Bd(x,y) \text { by (3.24)}} \nonumber \\  &   \quad + \overbrace{\left| \left( TP-I\right) \tilde{\phi }(x,d(x,y))-\lambda \right| }^{=0 \text { by (3.13)}} + \overbrace{\left| \phi (x,y)- \tilde{\phi }(x,d(x,y))\right| }^{= 0 \text { by (3.20)}}, \end{aligned}$$

for $$(x,y) \in \Delta _{R}\setminus \Delta $$. So

3.34 $$\begin{aligned} \left| \left( P_{(2)}-I\right) \phi (x,y) - \lambda \right| \le Bd(x,y), \end{aligned}$$

for $$(x,y) \in \Delta _{R}\setminus \Delta $$.

This basically means that $\left( P_{(2)}-I\right) \phi$ near the diagonal is close to $\lambda$. To obtain functions $\phi ^\pm$ so that $P_{(2)} - I$ applied to them, after subtracting $\lambda$, has a definite sign, we add suitable functions to $\phi$: we take $\phi ^\pm :\mathbb {T}^2\setminus \Delta \rightarrow \mathbb {R}$ of the form

3.35 $$\begin{aligned} \phi ^\pm (x,y) = \phi (x,y) \pm c_1 W_{-{q_0}}(x,y), \end{aligned}$$

for a small positive value $q_0\in (0,1/2)$, such that $\Lambda (q_0) \ne 0$, and $|c_1|$ large enough (will be defined below), with sign such that $c_1 (e^{\Lambda (q_0)}-1) > 0$. Note that $\phi ^\pm \in \mathcal {C}^0(\mathbb {T}^2\setminus \Delta , \mathbb {R})$.

By Proposition 3.2, we obtain

3.36 $$\begin{aligned} \left( TP-I\right) \tilde{W}_{-q_0}(x,u) = \left( e^{\Lambda (q_0)}-1\right) u^{q_0}\phi _{-q_0}(x), \end{aligned}$$

for $$(x,u) \in {\mathbb {T}\times \mathbb {R}^+}$$.

Combining (3.34), (3.36), (3.26) and (3.23) allows us to prove the first inequality of (3.31), by choosing $|c_1|$ in (3.35) large enough. For $(x,y) \in \Delta _R{\setminus } \Delta$,

3.37 $$\begin{aligned}  &   \left( P_{(2)}-I\right) \phi ^+(x,y) - \lambda = \left( P_{(2)}-I\right) \phi (x,y) - \lambda + c_1\left( P_{(2)}-I\right) W_{{-q_0}}(x,y) \nonumber \\  &   \quad = \overbrace{\left( P_{(2)}-I\right) \phi (x,y) - \lambda }^{\ge - B d(x,y) \text { by (3.34)}} + \overbrace{ c_1 (TP-I)\tilde{W}_{{-q_0}}(x,d(x,y))}^{\ge c_1 \left( e^{\Lambda (q_0)}-1\right) d(x,y)^{q_0}/C \text { by (3.36)}} \nonumber \\  &   \qquad + \overbrace{ c_1 \left( P_{(2)}W_{{-q_0}}(x,y)-TP\tilde{W}_{{-q_0}}(x,d(x,y))\right) }^{\ge - |c_1| B d(x,y)^{q_0+1} \text { by (3.26)} } \quad \nonumber \\  &   \qquad + \quad \overbrace{c_1 \left( W_{{-q_0}}(x,y)-\tilde{W}_{{-q_0}}(x,d(x,y)) \right) }^{=0 \text { by (3.23)}} \nonumber \\  &   \quad \ge - B d(x,y) + c_1\left( e^{\Lambda (q_0)}-1\right) d(x,y)^{q_0}/C - |c_1|B d(x,y)^{q_0+1}, \end{aligned}$$

so that

$$\begin{aligned} \left( P_{(2)}-I\right) \phi ^+(x,y) \ge \lambda , \end{aligned}$$

if *R* is small and $|c_1|$ is chosen large enough. The second inequality regarding $\phi ^-$ is obtained using similar bounds. The bound (3.30) for suitable $K>0$ is immediate from the expressions for $\phi ^\pm$.

We take $\lambda = 0$ and proceed with the construction of $\eta ^\pm$. We can bound $\left( P_{(2)}-I\right) \eta (x,y)$ by applying the triangle inequality and using (3.15), (3.25) and (3.21). This yields

$$\begin{aligned}  &   \left| \left( P_{(2)}-I\right) \eta (x,y)-V\right| \le \overbrace{\left| \left( TP\tilde{\eta }(x,d(x,y))-P_{(2)}\eta (x,y)\right) \right| }^{\le Bd(x,y)|\ln (d(x,y))| \text { by (3.15)} }+ \\  &   \quad \overbrace{\left| \left( TP-I\right) \eta (x,d(x,y))-V\right| }^{= 0 \text { by (3.25)} } + \overbrace{\left| \eta (x,y)- \tilde{\eta }(x,d(x,y))\right| }^{= 0 \text { by (3.21)} } \\ \end{aligned}$$

so that

3.38 $$\begin{aligned} \left| \left( P_{(2)}-I\right) \eta (x,y)-V\right|&\le Bd(x,y)|\ln (d(x,y))|, \end{aligned}$$

for all $(x,y) \in \Delta _{R}\setminus \Delta .$ We may not have that $\left( P_{(2)}-I\right) \eta (x,y) - V$ has a definite sign. We therefore add functions to $\eta$ (as we did to obtain $\phi ^\pm$ from $\phi$) and let

3.39 $$\begin{aligned} \eta ^\pm (x,y) = \eta (x,y) \pm c_2 W_{{-q_0}}(x,d(x,y)), \end{aligned}$$

with $|c_2|$ large enough (will be defined below), with sign such that $c_2 (e^{\Lambda (q_0)}-1) > 0$. We clearly have that $\eta ^\pm \in \mathcal {C}^0(\mathbb {T}^2\setminus \Delta , \mathbb {R})$.

Combining (3.15),(3.26) and (3.36) allows us to prove the first inequality of (3.33), by choosing $c_2$ in (3.39) large enough. For $(x,y) \in \Delta _R\setminus \Delta$, we get through a straightforward combination of previous inequalities,

$$\begin{aligned}  &   \left( P_{(2)}-I\right) \eta ^+(x,y) \\  &   \quad = \overbrace{\left( P_{(2)}-I\right) \eta (x,y)}^{\ge V -Bd(x,y)|\ln (d(x,y))| \text { by (3.38)}} + \overbrace{c_2 \left( P_{(2)}-I\right) W_{{-q_0}}(x,y),}^{\ge c_2\left( e^{\Lambda (q_0)}-1\right) d(x,y)^{q_0}/C - |c_2| B d(x,y)^{q_0+1} \text { by (3.37)}} \end{aligned}$$

so that

$$\begin{aligned} \left( P_{(2)}-I\right) \eta ^+(x,y) \ge V, \end{aligned}$$

for *R* small enough, by choosing $c_2$ large enough. Here we use the fact that for $0<R<1$, and $q_0\in (0,1)$, there exists a $C>0$, such that for all $x \in (0,R)$, $Cx^{q_0} > -x\ln (x)$. The second inequality in (3.33) for $\eta ^-$ is obtained using similar bounds.

Finally, (3.32) for *K* large enough is clear from the expression for $\eta ^\pm$, using Proposition 3.2. $\square$

#### Remark 3.15

We can use (3.31) to get

$$\begin{aligned} \pm \mathbb E\left[ \phi ^+(T_\omega (x),T_\omega (y)) \right] -\lambda \ge \pm \phi ^\pm (x,y), \end{aligned}$$

for *d*(*x*, *y*) small enough. Similarly if we assume $\lambda =0$, we get

$$\begin{aligned} \pm \mathbb E\left[ \eta ^+(T_\omega (x),T_\omega (y)) \right] -V \ge \pm \eta ^\pm (x,y), \end{aligned}$$

for *d*(*x*, *y*) small enough. Applying Doob’s stopping time theorem (we refer to standard references such as [63] or [32]) with suitable stopping times we build sub- and supermartingales for such random sequences from the functions $\phi ^+$, $\phi ^-$, $\eta ^+$ and $\eta ^-$. This is done in the proof of Lemma 4.1. $\square$

#### Proposition 3.16

There exists a $R,K>0$, and a family of continuous functions,

$$\begin{aligned} W_q^\pm \in \mathcal {C}^0(\mathbb {T}^2\setminus \Delta , \mathbb {R}),\end{aligned}$$

for $q \in [-|\gamma | - 1/2, |\gamma |+1/2]$, such that the following holds for all $(x,y) \in \Delta _R\setminus \Delta$.

For $W_q^\pm$ we have

3.40 $$\begin{aligned} \frac{1}{K} d(x,y)^{-q} \le W^\pm _q(x,y)&\le K d(x,y)^{-q}, \end{aligned}$$

3.41 $$\begin{aligned} \left( P_{(2)}-e^{\Lambda (-q)}\right) W_q^- (x,y)&\le 0 \le \left( P_{(2)}-e^{\Lambda (-q)}\right) W_q^+(x,y). \end{aligned}$$

#### Proof

We can bound $\left( P_{(2)}-e^{\Lambda (-q)}\right) W_q (x,y)$, by applying the triangle inequality and using Lemma 3.13:

$$\begin{aligned}  &   \left| \left( P_{(2)}-e^{\Lambda (-q)}\right) W_q (x,y)\right| \le \\  &   \quad \overbrace{ \left| P_{(2)}W_q (x,y) -TP\tilde{W}_q(x,d(x,y)) \right| }^{\le B d(x,y)^{-q+1} \text { by (3.26)}} + \overbrace{\left| \left( TP-e^{\Lambda (-q)}\right) \tilde{W}_q(x,d(x,y))\right| }^{= 0 \text { by (3.11)}}\\  &   \quad + e^{\Lambda (-q)} \overbrace{\left| W_q(x,y)- \tilde{W}_q(x,d(x,y))\right| }^{= 0 \text { by (3.23)}}, \end{aligned}$$

for $$(x,y) \in \Delta _{R}\setminus \Delta $$. So

3.42 $$\begin{aligned} \left| \left( P_{(2)}-e^{\Lambda (-q)}\right) W_q (x,y)\right| \le B d(x,y)^{-q+1}, \end{aligned}$$

for $$(x,y) \in \Delta _{R}\setminus \Delta $$.

This basically means that $\left( P_{(2)}-e^{\Lambda (-p)}\right) W_q$ near the diagonal is close to 0. To obtain functions $W_q^\pm$ so that $P_{(2)} - e^{\Lambda (-p)}$ applied to them, has a definite sign, we add suitable functions to $W_q$: we take $W_q^\pm :T^2\setminus \Delta \rightarrow \mathbb {R}$ of the form

$$\begin{aligned} W_q^\pm (x,y) = W_q(x,y) \pm c_3 W_{{q_1}}(x,y), \end{aligned}$$

for $q_1 \in (q-1/2, q)$, such that $\Lambda (-q) - \Lambda (-q_1) \ne 0$, and $|c_3|$ large enough (will be defined below), with sign such that $c_3 (e^{\Lambda (-q)} - e^{\Lambda (-q_1)}) >0.$

By applying (3.42) we get for $(x,y) \in \Delta _R\setminus \Delta$,

$$\begin{aligned}  &   \left( P_{(2)}-e^{\Lambda (-q)}\right) W_q^+ (x,y)= \\  &   \quad \overbrace{\left( P_{(2)}-e^{\Lambda (-q)}\right) W_q(x,y)}^{\ge -B d(x,y)^{-q+1} \text { by (3.42)} } + \overbrace{c_3 \left( P_{(2)}-e^{\Lambda (-q_1)}\right) W_{{q_1}}(x,y)}^{\ge -c_3\left( B d(x,y)^{-q_1+1}\right) \text { by (3.42)}}\\  &   \quad \quad + \overbrace{c_3 (e^{\Lambda (-q_1)}-e^{\Lambda (-q)})W_{q_1}.}^{\ge c_3(e^{\Lambda (-q_1)}-e^{\Lambda (-q)})d(x,y)^{-q_1}/K \text { by (3.22)}} \end{aligned}$$

Picking $c_3$ large enough and *R* small enough yields,

$$\begin{aligned} \left( P_{(2)}-e^{\Lambda (-q)}\right) W_q^+ (x,y) \ge 0, \quad \text { for } (x,y) \in \Delta _R\setminus \Delta . \end{aligned}$$

Similarly we get

$$\begin{aligned} \left( P_{(2)}-e^{\Lambda (-q)}\right) W_q^- (x,y) \le 0, \quad \text { for } (x,y) \in \Delta _R\setminus \Delta . \end{aligned}$$

Now set *K* again large enough such that (3.40) holds.

#### Remark 3.17

Suppose $\gamma$ is such that $e^{\Lambda (\gamma )} = 1$. We can use (3.41) to get

$$\begin{aligned} \pm \mathbb E\left[ W_{-\gamma }^\pm (T_\omega (x),T_\omega (y)) \right] \ge \pm W_{-\gamma }^\pm (x,y), \end{aligned}$$

for *d*(*x*, *y*) small enough. Applying Doob’s stopping time theorem with suitable stopping times we build sub- and supermartingales for such random sequences from the functions $W^+_\gamma$ and $W^-_\gamma$. This is done in the proof of Lemma 4.5.

## Topological Random Dynamics

In this section we prove Theorem 1.1 and construct tools to prove Theorem 1.2 in Sect. 5, for $\lambda \ge 0$. We look separately at cases with zero Lyapunov exponent, positive Lyapunov exponent and negative Lyapunov exponent.

### Zero Lyapunov exponent

The next part of the analysis is to calculate escape probabilities and expected escape times for escape from neighborhoods of the diagonal and strips near the diagonal. The proofs in this section rely on an analysis of Koopman operators, which is developed in Sect. 3. The statements can be read without reference to Sect. 3, but for the proofs the reader has to familiarize with the results in Sect. 3.

In order study escapes from strips near the diagonal, we define stopping times

4.1 $$\begin{aligned} \tau _{\delta ,+}(x,y)&:=\min \{n\in \mathbb N \; ; \; d(T_\omega ^n(x), T_\omega ^n(y))> \delta \}, \end{aligned}$$

4.2 $$\begin{aligned} \tau _{\varepsilon ,-}(x,y)&:=\min \{n\in \mathbb N \; ; \; d(T_\omega ^n(x), T_\omega ^n(y))< \varepsilon \}, \end{aligned}$$

where $(x,y) \in \Delta _R$ with $0<\varepsilon< d(x,y)< \delta <R$. The following lemma addresses statistics of these stopping times.

#### Lemma 4.1

For $\lambda = 0$, there exists a sufficiently small $R>0$, and sufficiently large $K>0$, such that if $0<\varepsilon<d(x,y)<\delta < R$, then

4.3 $$\begin{aligned} \mathbb {P}(\min \{\tau _{\varepsilon ,-}(x,y), \tau _{\delta ,+}(x,y)\}< \infty ) = 1, \end{aligned}$$

as well as

4.4 $$\begin{aligned} \frac{\ln \left( \frac{\delta }{d(x,y)}\right) -2K}{\ln \left( \frac{\delta }{\varepsilon }\right) } \le \mathbb {P}\left( \{\tau _{\varepsilon ,-}(x,y)< \tau _{\delta ,+}(x,y)\right) \} \le \frac{\ln \left( \frac{\delta }{d(x,y)}\right) +2K}{\ln \left( \frac{\delta }{\varepsilon }\right) } \end{aligned}$$

and

4.5 $$\begin{aligned}  &   \frac{1}{V} \left( \ln \left( \frac{\delta }{d(x,y)} \right) \ln \left( \frac{d(x,y)}{\varepsilon } \right) - 6K |\ln (\varepsilon )| -2K^2 \right) \nonumber \\  &   \quad \le \mathbb {E} \left[ \min \{\tau _{\varepsilon ,-}(x,y), \tau _{\delta ,+}(x,y)\}\right] \nonumber \\  &   \quad \le \frac{1}{V} \left( \ln \left( \frac{\delta }{d(x,y)} \right) \ln \left( \frac{d(x,y)}{\varepsilon } \right) + 6K |\ln (\varepsilon )| + 2K^2 \right) . \end{aligned}$$

Note that the estimates (4.4) and (4.5) are only informative when both $\delta /d(x,y)$ and $d(x,y)/\varepsilon$ are sufficiently large.

#### Proof

We fix $R>0$ small enough and $K>0$ large enough such that Proposition 3.14 and Proposition 3.16 hold simultaneously. We follow the reasoning of [11, 13]. As indicated above, we will use statements from Sect. 3. The functions $\phi ^\pm$ and $\eta ^\pm$ come directly from Proposition 3.14. Denote $(x_n,y_n) = T_\omega ^n(x_0,y_0),$ for $(x_0,y_0)\in \mathbb {T}^2{\setminus }\Delta$, such that $0<\varepsilon<d(x,y)<\delta < R$. Now $\eta ^+(x_n,y_n)$ stopped at $\min \{\tau _{\varepsilon ,-}(x,y), \tau _{\delta ,+}(x,y)\}$ is a submartingale, by Proposition 3.14, Remark 3.15 and applying Doob’s stopping time theorem. So, with

$$\begin{aligned} \tilde{n} = \min \{n, \tau _{\varepsilon ,-}(x,y), \tau _{\delta ,+}(x,y)\}, \end{aligned}$$

we have, for a fixed $n\in \mathbb {N}$, by Doob’s stopping time theorem with bounded stopping time,

$$\begin{aligned} \eta ^+(x,y) \le \mathbb {E} \left[ \eta ^+(x_{\tilde{n}},y_{\tilde{n}})-\tilde{n}V\right] . \end{aligned}$$

Rewriting, we obtain

$$\begin{aligned} \mathbb E[\tilde{n}] V&\le \mathbb {E} \left[ \eta ^+(x_{\tilde{n}},y_{\tilde{n}})\right] - \eta ^+(x,y)\\&\le \ln ^2(d(x,y))+\ln ^2(\varepsilon a_1) + K|\ln (\varepsilon a_1)|<\infty . \end{aligned}$$

Taking $n\rightarrow \infty$ and by the monotone convergence theorem, we have

$$\begin{aligned}\mathbb E[\min \{\tau _{\varepsilon ,-}(x,y), \tau _{\delta ,+}(x,y)\}] <\infty .\end{aligned}$$

This implies (4.3).

By Proposition 3.14 and Remark 3.15 and applying Doob’s stopping time theorem now for almost surely bounded stopping time, $\phi ^+(x_n,y_n)$ stopped at $\min \{\tau _{\varepsilon ,-}(x,y), \tau _{\delta ,+}(x,y)\}$ is a submartingale. Therefore we have

$$\begin{aligned} \phi ^+(x,y) \le \mathbb {E} \left[ \phi ^+(x_{\tilde{n}},y_{\tilde{n}})\right] . \end{aligned}$$

Letting *n* go to infinity, applying dominated convergence theorem, and conditioning separately on $\tau _{\varepsilon ,-}(x,y)< \tau _{\delta ,+}(x,y)$ or $\tau _{\varepsilon ,-}(x,y)< \tau _{\delta ,+}(x,y)$, we get

4.6 $$\begin{aligned}  &   \phi ^+(x,y) \le \lim _{ n\rightarrow \infty } \mathbb {E} \left[ \phi ^+(x_{\tilde{n}},y_{\tilde{n}})\right] \nonumber \\  &   \quad = \mathbb {P} \left( \tau _{\varepsilon ,-}(x,y)< \tau _{\delta ,+}(x,y)\right) \mathbb {E} \left[ \phi ^+(x_{\tau _{\varepsilon ,-}(x,y)},y_{\tau _{\varepsilon ,-}(x,y)})\mid \tau _{\varepsilon ,-}(x,y)< \tau _{\delta ,+}(x,y)\right] \nonumber \\  &   \qquad + \mathbb {P} \left( \tau _{\varepsilon ,-}(x,y)> \tau _{\delta ,+}(x,y)\right) \mathbb {E} \left[ \phi ^+(x_{\tau _{\delta ,+}(x,y)},y_{\tau _{\delta ,+}(x,y)})\mid \tau _{\varepsilon ,-}(x,y)> \tau _{\delta ,+}(x,y)\right] . \nonumber \\ \end{aligned}$$

By (4.3) we have

4.7 $$\begin{aligned} P \left( \tau _{\varepsilon ,-}(x,y)> \tau _{\delta ,+}(x,y)\right)&= 1 - \mathbb {P} \left( \tau _{\varepsilon ,-}(x,y)< \tau _{\delta ,+}(x,y)\right) . \end{aligned}$$

From (3.30) we get

4.8 $$\begin{aligned} \ln (d(x,y)) - K&\le \phi ^+(x,y). \end{aligned}$$

On the iterate *n* when the distance $d(x_{n},y_{n})$ first leaves $(\varepsilon ,\delta )$, the distance is either in $(\varepsilon a_1,\varepsilon ]$ or in $[\delta , \delta a_2)$ by Hypothesis (H1). Therefore we can bound the conditional expectation in (4.6) in the following manner,

4.9 $$\begin{aligned}  &   \mathbb {E} \left[ \phi ^+(x_{\tau _{\varepsilon ,-}(x,y)},y_{\tau _{\varepsilon ,-}(x,y)})\mid \tau _{\varepsilon ,-}(x,y)< \tau _{\delta ,+}(x,y)\right] \le \nonumber \\  &   \sup _{r \in [a_1\varepsilon ,\varepsilon )} \{\ln (r) + K\} \le \ln (\varepsilon ) + K \end{aligned}$$

and

4.10 $$\begin{aligned}  &   \mathbb {E} \left[ \phi ^+(x_{\tau _{\delta ,+}(x,y)},y_{\tau _{\delta ,+}(x,y)})\mid \tau _{\varepsilon ,-}(x,y)> \tau _{\delta ,+}(x,y)\right] \le \nonumber \\  &   \quad \sup _{r \in [\delta ,a_2\delta ]} \{\ln (r) + K\}\le \ln (\delta ) + 2K, \end{aligned}$$

for *K* chosen large enough. Using the bounds (4.8), (4.94.10), (4.94.10) and the equality (4.7) in (4.6), we get

$$\begin{aligned} \mathbb {P} \left( \tau _{\varepsilon ,-}(x,y)< \tau _{\delta ,+}(x,y)\right)&\le \frac{\displaystyle \ln \left( \frac{d(x,y)}{ \delta }\right) -4K}{\displaystyle \ln \left( \frac{\varepsilon }{ \delta }\right) }. \end{aligned}$$

In a similar fashion we get the counterpart of (4.6) for $\phi ^-$,

$$\begin{aligned}  &   \phi ^-(x,y) \ge \lim _{n\rightarrow \infty } \mathbb {E} \left[ \phi ^-(x_{\tilde{n}},y_{\tilde{n} })\right] \\  &   \quad = \mathbb {P} \left( \tau _{\varepsilon ,-}(x,y)< \tau _{\delta ,+}(x,y)\right) \mathbb {E} \left[ \phi ^-(x_{\tau _{\varepsilon ,-}(x,y)},y_{\tau _{\varepsilon ,-}(x,y)})\mid \tau _{\varepsilon ,-}(x,y)< \tau _{\delta ,+}(x,y)\right] \\  &   \qquad + \mathbb {P} \left( \tau _{\varepsilon ,-}(x,y)> \tau _{\delta ,+}(x,y)\right) \mathbb {E} \left[ \phi ^-(x_{\tau _{\delta ,+}(x,y)},y_{\tau _{\delta ,+}(x,y)})\mid \tau _{\varepsilon ,-}(x,y)> \tau _{\delta ,+}(x,y)\right] , \end{aligned}$$

from which we obtain a bound

4.11 $$\begin{aligned} \mathbb {P} \left( \tau _{\varepsilon ,-}(x,y)< \tau _{\delta ,+}(x,y)\right)&\ge \frac{\displaystyle \ln \left( \frac{d(x,y)}{\delta }\right) +4K}{\displaystyle \ln \left( \frac{\varepsilon }{ \delta }\right) }, \end{aligned}$$

again assuming that *K* is chosen large enough. This finishes the proof of (4.4).

For (4.5) we use the submartingale property of $\eta ^+(x_n,y_n) { - nV}$ stopped at $\tau _{\varepsilon ,-}(x,y)< \tau _{\delta ,+}(x,y)$ or $\tau _{\varepsilon ,-}(x,y)< \tau _{\delta ,+}(x,y)$ (see Remark 3.15 and apply Doob’s stopping time theorem.) and we let time go to infinity. Denoting

$$\begin{aligned} \tau = \min \{\tau _{\delta ,+}(x,y), \tau _{\varepsilon ,-}(x,y)\}, \end{aligned}$$

this yields

$$\begin{aligned} \eta ^+(x,y) \le \mathbb {E} \left[ \eta ^+(x_\tau ,y_\tau ) -V \tau \right] . \end{aligned}$$

Rewriting and conditioning on $\tau _{\varepsilon ,-}(x,y)< \tau _{\delta ,+}(x,y)$ and $\tau _{\varepsilon ,-}(x,y)< \tau _{\delta ,+}(x,y)$ separately, we obtain

4.12 $$\begin{aligned}  &   V\mathbb {E} \left[ \tau \right] \le \mathbb {P}\left( \tau _{\varepsilon ,-}(x,y)< \tau _{\delta ,+}(x,y)\right) \mathbb {E} \left[ \eta ^+(x_\tau ,y_\tau )|\tau _{\varepsilon ,-}(x,y)< \tau _{\delta ,+}(x,y) \right] \nonumber \\  &   \quad + \mathbb {P}\left( \tau _{\varepsilon ,-}(x,y)> \tau _{\delta ,+}(x,y)\right) \mathbb {E} \left[ \eta ^+(x_\tau ,y_\tau )|\tau _{\varepsilon ,-}(x,y)> \tau _{\delta ,+}(x,y) \right] - \eta ^+(x,y).\nonumber \\ \end{aligned}$$

As above we have bounds

$$\begin{aligned} \mathbb {E} \left[ \eta ^+(x_\tau ,y_\tau )|\tau _{\varepsilon ,-}(x,y)< \tau _{\delta ,+}(x,y) \right]&\le \ln ^2(a_1 \varepsilon ) + K \left| \ln (a_1 \varepsilon ) \right| \\&\le \ln ^2(\varepsilon ) + 3K \left| \ln (\varepsilon ) \right| +2K^2, \\ \mathbb {E} \left[ \eta ^+(x_\tau ,y_\tau )|\tau _{\varepsilon ,-}(x,y)> \tau _{\delta ,+}(x,y) \right]&\le \ln ^2 (\delta ) + K \left| \ln (\delta ) \right| , \end{aligned}$$

Now for a different, larger *K*, we have $\mathbb {E} \left[ \eta ^+(x_\tau ,y_\tau )|\tau _{\varepsilon ,-}(x,y)< \tau _{\delta ,+}(x,y) \right] \le \ln ^2(\varepsilon )+ K|\log (\varepsilon )|$ and we have

$$\begin{aligned} -\eta ^+(x,y)&\le -\ln ^2(d(x,y)) + K \left| \ln (d(x,y)) \right| . \end{aligned}$$

Plugging this and (4.7), (4.11), (3.32) into the estimate (4.12), we obtain

$$\begin{aligned} V\mathbb {E} \left[ \tau \right]\le &   \frac{\displaystyle \ln \left( \frac{d(x,y)}{\delta }\right) +4K}{\displaystyle \ln \left( \frac{\varepsilon }{ \delta }\right) }\left( \ln ^2\left( {\varepsilon }\right) -\ln ^2\left( \delta \right) + K \left| \ln \left( \frac{\varepsilon }{\delta } \right) \right| \right) \\  &   + \ln ^2(\delta ) + 2K |\ln (\delta )| - \ln ^2(d(x,y)) + 2K |\ln (d(x,y))|. \end{aligned}$$

Note that $\ln ^2\left( {\varepsilon }\right) -\ln ^2\left( \delta \right) = \ln \left( \frac{\varepsilon }{\delta } \right) \ln (\varepsilon \delta )$ and $\ln ^2(\delta ) - \ln ^2(d(x,y)) = \ln \left( \frac{\delta }{d(x,y)}\right) \ln (d(x,y)\delta )$. As $|\ln (x)|$ is a decreasing function for $0<x<1$, we have $|\ln (\varepsilon )|< |\ln (d(x,y))| < |\ln (\delta )|$. Using these identities and estimates we get

$$\begin{aligned} V\mathbb {E} \left[ \tau \right]\le &   \ln \left( \frac{d(x,y)}{\delta }\right) \ln (\varepsilon \delta ) - \ln \left( \frac{d(x,y)}{\delta }\right) \ln \left( d(x,y)\delta \right) + 12K|\ln (\varepsilon )| + 8K^2 \\\le &   \ln \left( \frac{d(x,y)}{\delta }\right) \ln \left( \frac{\varepsilon }{d(x,y)}\right) + 12K|\ln (\varepsilon )| + 8K^2. \end{aligned}$$

In a similar fashion we get

$$\begin{aligned}  &   V\mathbb {E} \left[ \tau \right] \ge \frac{\displaystyle \ln \left( \frac{d(x,y)}{\delta }\right) -4K}{\displaystyle \ln \left( \frac{\varepsilon }{ \delta }\right) }\left( \ln ^2\left( \varepsilon \right) -\ln ^2\left( \delta \right) + 2K \left| \ln \left( \frac{\varepsilon }{\delta } \right) \right| \right) \\  &   \quad + \ln ^2(\delta ) - 2K |\ln (\delta )| - \ln ^2(d(x,y)) + 2K |\ln (d(x,y))|, \end{aligned}$$

and from this,

$$\begin{aligned} V\mathbb {E} \left[ \tau \right] \ge \ln \left( \frac{d(x,y)}{\delta }\right) \ln \left( \frac{\varepsilon }{d(x,y)}\right) - 12K|\ln (\varepsilon )| - 8K^2. \end{aligned}$$

Replace 2*K* by *K* to get the statement of the lemma. This finishes the proof.

From here on, *R*, *K* are fixed as in Lemma 4.1. We now formulate a result stating that trajectories of points $(x,y) \in \Delta _\delta$ will almost surely escape from $\Delta _\delta$. However, the expected escape time will be infinite for *d*(*x*, *y*) sufficiently small.

#### Lemma 4.2

Let *R*, *K* be as in Lemma 4.1. Then for all $(x,y) \in \mathbb {T}^2$, with $0<d(x,y)< \delta <R$,

4.13 $$\begin{aligned} \mathbb {P}\left( \tau _{\delta ,+}(x,y)< \infty \right) = 1 \end{aligned}$$

and

$$\begin{aligned} \mathbb {E} \left[ \tau _{\delta ,+}(x,y)\right] = \infty \text { whenever } d(x,y) < e^{-6K}\delta . \end{aligned}$$

#### Proof

For any $\varepsilon \in (0, d(x,y))$, we have the inclusion of events

$$\begin{aligned} \{ \tau _{\delta ,+}(x,y) \le \tau _{\varepsilon ,-}(x,y) \} \subset \{ \tau _{\delta ,+}(x,y) < \infty \}. \end{aligned}$$

Therefore,

$$\begin{aligned} \mathbb {P}(\tau _{\delta ,+}(x,y)< \infty ) \ge \mathbb {P}(\tau _{\delta ,+}(x,y) \le \tau _{\varepsilon ,-}(x,y)) = 1 - \mathbb {P} (\tau _{\varepsilon ,-}(x,y) < \tau _{\delta ,+} (x,y)). \end{aligned}$$

Recall the upper bound in (4.4),

$$\begin{aligned} \mathbb {P} (\tau _{\varepsilon ,-}(x,y) < \tau _{\delta ,+} (x,y)) \le \frac{\ln \left( \frac{\delta }{d(x,y)}\right) +2K}{\ln \left( \frac{\delta }{\varepsilon }\right) }. \end{aligned}$$

As $\varepsilon \rightarrow 0$, the denominator goes to infinity while the numerator remains fixed. Thus the term on the right hand side goes to 0, implying $\mathbb {P}(\tau _{\delta ,+}(x,y) < \infty ) = 1$. For the second statement, observe that for any $0< \varepsilon < d(x,y)$,

$$\begin{aligned} \tau _{\delta ,+} (x,y) \ge \min \{\tau _{\varepsilon ,-}(x,y), \tau _{\delta ,+}(x,y)\}. \end{aligned}$$

Taking expectations and applying the lower bound from (4.5) in Lemma 4.1, we find

$$\begin{aligned} V \mathbb {E}[\tau _{\delta ,+}(x,y)]&\ge V \mathbb {E}[\min \{\tau _{\varepsilon ,-}(x,y), \tau _{\delta ,+}(x,y)\}] \\  &\ge \ln \left( \frac{\delta }{d(x,y)} \right) \ln \left( \frac{d(x,y)}{\varepsilon } \right) - 6K | \ln (\varepsilon )| - 2K^2. \end{aligned}$$

Recalling that for small $\varepsilon$, $\ln (d(x,y)/\varepsilon ) = \ln (d(x,y)) + | \ln (\varepsilon )|$, we group the terms involving $|\ln (\varepsilon )|$:

$$\begin{aligned} V \mathbb {E}[\tau _{\delta ,+}(x,y)]&\ge \ln \left( \frac{\delta }{d(x,y)} \right) \left( \ln (d(x,y)) + |\ln (\varepsilon )| \right) - 6K |\ln (\varepsilon )| - 2K^2 \\  &= |\ln (\varepsilon )| \left[ \ln \left( \frac{\delta }{d(x,y)} \right) - 6K \right] + \ln \left( \frac{\delta }{d(x,y)} \right) \ln (d(x,y)) - 2K^2. \end{aligned}$$

The hypothesis $d(x,y) < e^{-6K}\delta$ gives $\ln \left( \frac{\delta }{d(x,y)} \right) - 6K > 0.$ Taking the limit $\varepsilon \rightarrow 0$ and applying the monotone convergence theorem, yields

$$\begin{aligned} \mathbb {E}[\tau _{\delta ,+}(x,y)] = \lim _{\varepsilon \rightarrow 0} \mathbb {E} [\tau _{\delta ,+}(x,y)\cdot 1\!\!1_{\{\tau _{\delta ,+}(x,y)< \tau _{\epsilon ,-}(x,y)\}}(\omega )] = \infty . \end{aligned}$$

$$\square $$

From this lemma we know that for $(x_0,y_0) \in \Delta _\delta$, the expected number of orbit points

$$\begin{aligned}(x_n,y_n) = \left( T^{(2)}_\omega \right) ^n (x_0,y_0) \end{aligned}$$

before escaping $\Delta _\delta$, with $(x_0,y_0) \in \Delta _{ e^{-6K}\delta }$, is infinite.

The above results allow to conclude Theorem 1.1(2).

#### Proposition 4.3

If $\lambda =0$, for all $(x,y) \in \mathbb {T}^2\setminus \Delta$,

$$\begin{aligned} \lim _{n\rightarrow \infty } \frac{1}{n}\sum _{i=0}^{n-1} d\left( T^i_\omega (x),T^i_\omega (y)\right) = 0 ~\text {and}~ \limsup _{n\rightarrow \infty }d\left( T^n_\omega (x), T^n_\omega (y)\right) > 0,~\mathbb {P}-\mathrm {a.s.} \end{aligned}$$

#### Proof

Let $\varepsilon >0$. For $(x,y) \in \mathbb {T}^2\setminus \Delta$ and $\omega \in \Sigma _\vartheta$, consider the empirical count of iterates in $\Delta _\varepsilon$,

$$\begin{aligned} N_\varepsilon (x,y,\omega ) = \lim _{n\rightarrow \infty }\frac{\#\{i\in \mathbb {N}, 0\le i \le n-1: d(T_\omega ^i(x),T_\omega ^i(y))<\varepsilon \}}{n}. \end{aligned}$$

We follow reasoning in [4], adapting to our setting. Let $(x_0,y_0)$ be a point outside $\Delta _\varepsilon$ and consider the orbit $(x_i,y_i) = \left( T_\omega ^i (x_0), T_\omega ^i (y_0)\right)$. For almost all $\omega \in \Sigma _\vartheta$ there is an infinite sequence of times $S_i$ and $T_i$, $i \ge 0$, with $0 = S_0< T_0< S_1< T_1 < \cdots$, such that for $j \ge 0$,

$$\begin{aligned} T_j&= \min \left\{ i> S_j \; : \; d(x_i,y_i) < \varepsilon e^{-6K} \right\} , \\ S_{j+1}&= \min \left\{ i> T_j \; : \; d(x_i,y_i) > \varepsilon \right\} . \end{aligned}$$

We can also assume that $d(x_i,y_i)$ is never exactly $\varepsilon$, as this holds for almost all $\omega \in \Sigma _\vartheta$. Write

$$\begin{aligned} \sigma _{j+1} = T_j - S_j, \qquad \tau _{j+1} = S_{j+1} - T_j. \end{aligned}$$

Given $(x_0,y_0)$ outside $\Delta _\varepsilon$, write

$$\begin{aligned} N_\varepsilon (n,\omega ) = \frac{\#\{i\in \mathbb {N}, 0\le i \le n-1: d(x_i,y_i)<\varepsilon \}}{n}. \end{aligned}$$

Let $N_\sigma (n)$ be the largest index *i* with $S_i \le n$ and let $N_\tau (n)$ be the largest index *i* with $T_i < n$. Consider, for definiteness, the case that $d(x_n,y_n) > \varepsilon$. Calculate

$$\begin{aligned} N_\varepsilon (n,\omega )&\ge \frac{1}{T_{N_\tau (n)}} \sum _{i=1}^{T_{N_{\tau (n)}-1}} \tau _i / (\sigma _i + \tau _i) \end{aligned}$$

The dependence of, for instance, $\sigma _i$ on the orbit point $(x_{S_i}, y_{S_i})$, makes that we can not apply the strong law of large numbers for independent random variables. However, the arguments that lead to the strong law of large numbers, see for instance [32, Section 2.5] and [28, 29], combined with the uniform bounds on the expected escape times $\sigma _i$ do give that for some $K>0$, for almost all $\omega \in \Sigma _\vartheta$,

$$\begin{aligned} \limsup _{n\rightarrow \infty } \frac{1}{n} \sum _{i=0}^{n-1} \sigma _i&\le K. \end{aligned}$$

Likewise,

$$\begin{aligned} \liminf _{n\rightarrow \infty } \frac{1}{n} \sum _{i=0}^{n-1} \tau _i&= \infty , \end{aligned}$$

for almost all $\omega \in \Sigma _\vartheta$. The case that $d(x_n,y_n) > \varepsilon$ is treated similarly. We get that $\mathbb {P}$-almost surely $N_\varepsilon (x,y,\omega )=1$, for all $\varepsilon >0$ implying the first part of the statement.

The second part follows from Lemma 2.5. $\square$

### Positive Lyapunov exponent

In this section we consider positive Lyapunov exponent $\lambda > 0$. This will be assumed to hold throughout the section. Furthermore, we will denote the second zero of the moment Lyapunov function $\Lambda$ as $\gamma$.

Consider $0< \delta < R$ and $(x,y) \in \mathbb {T}^2{\setminus } \Delta$ with $d(x,y) < \delta$. Let $\tau (x,y,\omega )$ be the minimal time with

$$\begin{aligned} d\left( \left( T^{(2)}_\omega \right) ^{\tau (x,y,\omega )} (x,y) \right) > \delta , \end{aligned}$$

with $\tau (x,y,\omega )=\infty$ if this does not exist.

#### Lemma 4.4

Let *R*, *K* be as in Lemma 4.1. Suppose $\lambda > 0$. For $0< \delta < R$ and $(x,y) \in \mathbb {T}^2{\setminus } \Delta$ with $d(x,y) < \delta$ we have

$$\begin{aligned} \mathbb {P} \left( d(T^n_\omega (x), T^n_\omega (y)) > \delta \text { for some } n \in \mathbb {N} \right) = 1. \end{aligned}$$

Moreover, for some $K>0$,

4.14 $$\begin{aligned} \frac{1}{\lambda } \left( \ln \left( \frac{\delta }{d(x,y)} \right) - 2K \right) \le \mathbb {E}\left[ \tau (x,y,\omega ) \right] \le \frac{1}{\lambda } \left( \ln \left( \frac{\delta }{d(x,y)}\right) + 2K \right) . \end{aligned}$$

#### Proof

Write $x_n = T^n_\omega (x)$ and $y_n = T^n_\omega (x)$. We also use shorthand notation $\tau$ for $\tau (x,y,\omega )$. By Proposition 3.14 and Remark 3.15 we find, applying Doob’s stopping time theorem,

$$\begin{aligned} \lambda \mathbb {E} [\tau ] \le \mathbb {E} \left[ \phi ^+ (x_\tau ,y_\tau ) - \phi ^+ (x,y) \right] \le \ln (\delta ) - \ln (d(x,y)) + 2K \end{aligned}$$

and

$$\begin{aligned} \lambda \mathbb {E} [\tau ] \ge \mathbb {E} \left[ \phi ^- (x_\tau ,y_\tau ) - \phi ^- (x,y) \right] \ge \ln (\delta ) - \ln (d(x,y)) - 2K. \end{aligned}$$

The lemma follows. $\square$

More detailed estimates can be obtained by analysing escape times from strips $\Delta _\delta \setminus \Delta _\varepsilon$. The next lemma is the equivalent of Lemma 4.1 for positive Lyapunov exponent. Stopping times $\tau _{\delta ,+}(x,y)$ and $\tau _{\varepsilon ,-}(x,y)$ are defined as before in (4.14.2), (4.14.2).

#### Lemma 4.5

Let *R*, *K* be as in Lemma 4.1. Suppose $\lambda > 0$. Let $\gamma \ne 0$ be the negative value so that $\Lambda (\gamma ) = 0$. If $0<\varepsilon<d(x,y)<\delta < R$, then

4.15 $$\begin{aligned} \mathbb {P}(\min \{\tau _{\varepsilon ,-}(x,y), \tau _{\delta ,+}(x,y)\}< \infty ) = 1. \end{aligned}$$

Furthermore, there exists a $\kappa \in (0,1)$, such that if $0<\varepsilon<d(x,y)<\kappa \delta <\kappa R$, then

4.16 $$\begin{aligned} \frac{1}{K}\left( \frac{d(x,y)}{\varepsilon }\right) ^{\gamma } \le \mathbb {P}\left( \{\tau _{\varepsilon ,-}(x,y)< \tau _{\delta ,+}(x,y)\}\right) \le {K}\left( \frac{d(x,y)}{\varepsilon }\right) ^{\gamma }. \end{aligned}$$

#### Proof

The proof is similar to the proof of Lemma 4.1. Let $(x,y) \in \Delta _{\delta }\setminus \Delta _\varepsilon$, so with $\varepsilon< d(x,y) < \delta$. Let $\tau _{\varepsilon ,-}$ and $\tau _{\delta ,+}$ as in (4.14.2) and (4.14.2). As before we get (4.15), that is, $\min \{\tau _{\varepsilon ,-},\tau _{\delta ,+}\}<\infty$ for almost all $\omega$.

We start with the upper bound in (4.16). Using Remark 3.17 and applying Doob’s stopping time theorem, we get

4.17 $$\begin{aligned}  &   W^-_{-\gamma }(x,y) \ge \nonumber \\  &   \quad \mathbb {P} \left( \tau _{\varepsilon ,-}(x,y)< \tau _{\delta ,+}(x,y)\right) \mathbb {E} \left[ W^-_{-\gamma }(x_{\tau _{\varepsilon ,-}(x,y)},y_{\tau _{\varepsilon ,-}(x,y)})\mid \tau _{\varepsilon ,-}(x,y)< \tau _{\delta ,+}(x,y)\right] \nonumber \\  &   \qquad + \mathbb {P} \left( \tau _{\varepsilon ,-}(x,y)> \tau _{\delta ,+}(x,y)\right) \mathbb {E} \left[ W^-_{-\gamma }(x_{\tau _{\delta ,+}(x,y)},y_{\tau _{\delta ,+}(x,y)})\mid \tau _{\varepsilon ,-}(x,y)> \tau _{\delta ,+}(x,y)\right] . \nonumber \\ \end{aligned}$$

As before, since $\min \{\tau _{\varepsilon ,-},\delta \}$ is almost surely finite (see Lemma 4.1), we have

$$\begin{aligned} \mathbb {P} \left( \tau _{\varepsilon ,-}(x,y)> \tau _{\delta ,+}(x,y)\right)&= 1 - \mathbb {P} \left( \tau _{\varepsilon ,-}(x,y)< \tau _{\delta ,+}(x,y)\right) . \end{aligned}$$

Furthermore, for some $K>1$ as in Proposition 3.16, we get

$$\begin{aligned} \mathbb {E} \left[ W^-_{-\gamma }(x_{\tau _{\varepsilon ,-}(x,y)},y_{\tau _{\varepsilon ,-}(x,y)})\mid \tau _{\varepsilon ,-}(x,y)< \tau _{\delta ,+}(x,y)\right]&\ge \frac{\varepsilon ^{\gamma }}{K}, \\ \mathbb {E} \left[ W^-_{-\gamma }(x_{\tau _{\delta ,+}(x,y)},y_{\tau _{\delta ,+}(x,y)})\mid \tau _{\varepsilon ,-}(x,y)> \tau _{\delta ,+}(x,y)\right]&\ge \frac{\delta ^{\gamma }}{K}, \end{aligned}$$

and also $K d(x,y)^{\gamma } \ge W^-_{-\gamma }(x,y)$. Then (4.17) yields

$$\begin{aligned} \mathbb {P} \left( \tau _{\varepsilon ,-}(x,y) < \tau _{\delta ,+}(x,y)\right)&\le \left( {K^2} d(x,y)^{\gamma } - \delta ^{\gamma } \right) / \left( \varepsilon ^{\gamma } - \delta ^{\gamma } \right) . \end{aligned}$$

From this and a condition $d(x,y) \le \kappa \delta$ for small enough $\kappa$, we get the stated bound. The lower bound in (4.16) is derived similarly. $\square$

Using the above lemma we get the following statement in which we discuss, for orbit pieces starting in $\Delta _{\kappa \delta }\setminus \Delta _\varepsilon$ until escape from $\Delta _\delta$, the expected number of points in $\Delta _\varepsilon$. It is similar to Lemma 5.3, but for positive Lyapunov exponent.

The results in this section easily allow to conclude Theorem 1.1(3).

#### Proposition 4.6

If $\lambda > 0$, for all $(x,y) \in \mathbb {T}^2\setminus \Delta$,

$$\begin{aligned} \lim _{n\rightarrow \infty }\frac{1}{n}\sum _{i=0}^{n-1} d \left( T^i_\omega (x), T^i_\omega (y)\right) > 0 ~\text {and}~ \liminf _{n\rightarrow \infty }d\left( T^n_\omega (x),T^n_\omega (y)\right) = 0,~\mathbb {P}-\mathrm {a.s.} \end{aligned}$$

#### Proof

The reasoning follows Proposition 4.3. Let $\varepsilon >0$. For $(x,y) \in \mathbb {T}^2\setminus \Delta$ and $\omega \in \Sigma _\vartheta$, consider the empirical count of iterates in $\Delta _\varepsilon$,

$$\begin{aligned} N_\varepsilon (x,y,\omega ) = \lim _{n\rightarrow \infty }\frac{\#\{i\in \mathbb {N}, 0\le i \le n-1: d(T_\omega ^i(x),T_\omega ^i(y))<\varepsilon \}}{n}. \end{aligned}$$

By Lemmas 4.4 and 2.3 and the strong law of large numbers, we get that $\mathbb {P}$-almost surely $N_\varepsilon (x,y,\omega )<1$, for a $\varepsilon >0$ implying the first part of the statement. The second part follows from Lemma 2.5. $\square$

### Negative Lyapunov exponent

In this section we consider negative Lyapunov exponent $\lambda <0$. This will be assumed to hold throughout the section. The following lemma can be obtained by the construction of local stable manifolds for $T^n_\omega$ of points in $\Delta$, which exists for almost all $\omega \in \Sigma _\vartheta$ [49]. We provide a proof along the lines of our reasoning in previous sections, compare also [10].

#### Lemma 4.7

Let *R*, *K* be as in Lemma 4.1. Suppose $\lambda < 0$. There is $0< \chi <1$ so that the following holds. For each $0< \delta < R$ there is $0< \delta ' < \delta$, so that for (*x*, *y*) with $d(x,y) < \delta '$,

$$\begin{aligned} \mathbb {P} \left( d( T^n_\omega (x) , T^n_\omega (y)) < \delta \text { for all } n \text { and } \lim _{n\rightarrow \infty } d( T^n_\omega (x) , T^n_\omega (y)) = 0 \right)&> \chi . \end{aligned}$$

#### Proof

Let $\gamma >0$ given by Lemma 3.4 be so that $\Lambda (\gamma )=0$. The first part of the proof is similar to the proof of Lemmas 4.1 and  4.5. Take $(x,y) \in \Delta _{\delta }{\setminus } \Delta _\varepsilon$, so with $\varepsilon \le d(x,y) < \delta$. Let $\tau _{\varepsilon ,-}(x,y)$ and $\tau _{\delta ,+}(x,y)$ as in (4.14.2) and (4.14.2). As before we get (4.15), that is, $\min \{\tau _{\varepsilon ,-}(x,y),\tau _{\delta ,+}(x,y)\}<\infty$ for almost all $\omega$. Thus, as in Lemma 4.1,

4.18 $$\begin{aligned} \mathbb {P} \left( \tau _{\varepsilon ,-}(x,y)> \tau _{\delta ,+}(x,y)\right)&= 1 - \mathbb {P} \left( \tau _{\varepsilon ,-}(x,y)< \tau _{\delta ,+}(x,y)\right) . \end{aligned}$$

Using Remark 3.17 and applying Doob’s stopping time theorem, we get

4.19 $$\begin{aligned}  &   W^-_{-\gamma }(x,y) \ge \nonumber \\  &   \quad \mathbb {P} \left( \tau _{\varepsilon ,-}(x,y)< \tau _{\delta ,+}(x,y)\right) \mathbb {E} \left[ W^-_{-\gamma }(x_{\tau _{\varepsilon ,-}(x,y)},y_{\tau _{\varepsilon ,-}(x,y)})\mid \tau _{\varepsilon ,-}(x,y)< \tau _{\delta ,+}(x,y)\right] \nonumber \\  &   \qquad + \mathbb {P} \left( \tau _{\varepsilon ,-}(x,y)> \tau _{\delta ,+}(x,y)\right) \mathbb {E} \left[ W^-_{-\gamma }(x_{\tau _{\delta ,+}(x,y)},y_{\tau _{\delta ,+}(x,y)})\mid \tau _{\varepsilon ,-}(x,y)> \tau _{\delta ,+}(x,y)\right] . \nonumber \\ \end{aligned}$$

For some $K>1$ as in Proposition 3.16, we get

$$\begin{aligned} \mathbb {E} \left[ W^-_{-\gamma }(x_{\tau _{\varepsilon ,-}(x,y)},y_{\tau _{\varepsilon ,-}(x,y)})\mid \tau _{\varepsilon ,-}(x,y)< \tau _{\delta ,+}(x,y)\right]&\ge \frac{\varepsilon ^\gamma }{K}, \\ \mathbb {E} \left[ W^-_{-\gamma }(x_{\tau _{\delta ,+}(x,y)},y_{\tau _{\delta ,+}(x,y)})\mid \tau _{\varepsilon ,-}(x,y)> \tau _{\delta ,+}(x,y)\right]&\ge \frac{\delta ^\gamma }{K}, \end{aligned}$$

and also ${K} d(x,y)^{\gamma } \ge W^-_{-\gamma }(x,y)$. Using (4.18), (4.19) yields

4.20 $$\begin{aligned} \lim _{\varepsilon \rightarrow 0} \mathbb {P} \left( \tau _{\varepsilon ,-}(x,y)< \tau _{\delta ,+}(x,y)\right)&\ge \lim _{\varepsilon \rightarrow 0} \left( {K^2} d(x,y)^\gamma - \delta ^\gamma \right) / \left( \varepsilon ^\gamma - \delta ^\gamma \right) \nonumber \\&= 1 - {K^2} \left( \frac{d(x,y) }{ \delta } \right) ^\gamma . \end{aligned}$$

The limit exists because the probability is monotone decreasing as $\varepsilon \rightarrow 0$. This lower bound for $\mathbb {P}$ is strictly positive if $d(x,y) / \delta$ is small enough. The computation means that there is a strictly positive probability

$$\begin{aligned} \mathbb {P} \left( d(x_n,y_n) < \delta \text { for all } n\in \mathbb {N} \text { and } \liminf _{n\rightarrow \infty } d(x_n,y_n) = 0 \right) \ge 1 - {K^2} \left( \frac{d(x,y) }{ \delta } \right) ^\gamma , \end{aligned}$$

at least for $d(x,y)/\delta$ small enough. Note that $\mathbb {P}$ goes to one if $d(x,y) / \delta$ goes to zero.

We obtain the lemma from the observation that the above argument applies to any $\delta$. To finish the argument, take $0< d_2< \delta _2< d(x,y) < \delta$ and, using (4.20), consider

$$\begin{aligned}  &   \mathbb {P} \left( \tau _{d_2,-}(x,y)< \tau _{\delta ,+}(x,y) \text { and } d(x_i,y_i)< \delta _2 \text { for all } i \ge \tau _{d_2,-}(x,y) \right) \\  &   \quad \ge \left( 1 - {K^2} \left( \frac{d(x,y) }{ \delta } \right) ^\gamma \right) \left( 1 - {K^2} \left( \frac{d_2}{ \delta _2} \right) ^\gamma \right) . \end{aligned}$$

The lemma follows by taking $d_2$ and $\delta _2$ to zero, with $d_2/\delta _2$ small enough, and noting that the bound on the right hand side stays strictly positive. $\square$

Synchronisation of typical orbits expressed by Theorem 1.1(1) is a consequence of the above lemma and our hypotheses on the random dynamical system.

#### Proposition 4.8

Suppose $\lambda < 0$. For all $x,y \in \mathbb {T}$, for $\mathbb {P}$-almost all $\omega \in {\Sigma _\vartheta }$,

$$\begin{aligned} \lim _{n\rightarrow \infty } d(T^n_\omega (x),T^n_\omega (y)) = 0. \end{aligned}$$

#### Proof

Take $0< \delta ' < \delta$ as in Lemma 4.7. By Lemma 2.5 there is a strictly positive probability for an orbit $T^n_\omega (x,y)$ to enter $\Delta _{\delta '}$ in finitely many steps. That is, there exists $C>0$ and $N>0$ so that for $(x,y) \in \mathbb {T}^2 \setminus \Delta _{\delta '}$,

$$\begin{aligned} \mathbb {P} \left( (T^n_\omega (x), T^n_\omega (y)) \in \Delta _{\delta '} \text { for some } 0< n < N \right) > C. \end{aligned}$$

We find that for $(x,y) \in \mathbb {T}^2 {\setminus } \Delta _{\delta '}$,

$$\begin{aligned} \mathbb {P} \left( (T^n_\omega (x), T^n_\omega (y)) \not \in \Delta _{\delta '} \text { for all } 0 \le n < kN \right) \ge (1-C)^k, \end{aligned}$$

so that $\mathbb {P} \left( (T^n_\omega (x), T^n_\omega (y)) \not \in \Delta _{\delta '} \text { for all } n \right) = 0$. There are therefore almost surely infinitely many orbit points from $(T^n_\omega (x), T^n_\omega (y))$ in $\Delta _{\delta '}$.

As before, for $0< \varepsilon < \delta$ and $(x,y) \in \mathbb {T}^2$ with $\varepsilon< d(x,y) < \delta$, $(T^n_\omega (x), T^n_\omega (y))$ will be outside $\Delta _{\delta } {\setminus } \Delta _{\varepsilon }$ for some $n >0$, almost surely. Combined with the above we see that almost surely, orbit points $(T^n_\omega (x), T^n_\omega (y))$ are in $\Delta _\varepsilon$ infinitely often. This holds for any $\varepsilon >0$, so that

$$\begin{aligned} \liminf _{n\rightarrow \infty } d(T^n_\omega (x), T^n_\omega (y)) = 0 \end{aligned}$$

almost surely. That is, given a sequence $\varepsilon _k$ that is decreasing to zero, there is a sequence of first iterates $(T^{n_k}_\omega (x), T^{n_k}_\omega (y))$ inside $\Delta _{\varepsilon _k}$. Lemma 4.7 now implies synchronisation.

## Stationary Measures for the Two-Point Motion

This section contains both the construction of stationary measures on $\mathbb {T}^2\setminus \Delta$ for the random two-point maps, in the case of zero and positive Lyapunov exponent, and the derivation of their asymptotics at the diagonal. The results apply to zero and positive Lyapunov exponent.

### Construction by inducing

We construct a stationary Radon measure on $\mathbb {T}^2 {\setminus } \Delta$ for the two-point random dynamical system with Lyapunov exponent greater than or equal to zero. We do this by inducing: we construct a stationary measure for a first return map on a domain away from the diagonal $\Delta$ by the Krylov-Bogolyubov method. Following work by Deroin, Kleptsyn, Navas and Parwani [31], who study random walks on the real line, we introduce random stopping times in order to be able to apply a Krylov-Bogolyubov method to find stationary measures.

A stationary measure is obtained as usual by pushing forward the stationary measure for the first return map. For a zero Lyapunov exponent, Lemma 4.2 implies that it takes in expectation infinite time to get away from the diagonal and therefore the stationary measure is not finite. For a positive Lyapunov exponent, a stationary measure for the two-point motion can be constructed just as in the case of zero Lyapunov exponent. The finite expectation of the escape time from $\Delta _\delta$ as expressed by Lemma 4.4, shows that the total measure is finite. Normalizing the measure, we derive the existence of a stationary probability measure.

An alternative way to construct stationary measures for the positive Lyapunov exponent case $\lambda >0$, might be through a Lyapunov function as in [20] for random diffeomorphisms. Our case is different due to the possibility that points $(x,y)\in \mathbb {T}^2$ can be mapped onto the diagonal $\Delta$ by $T^{(2)}_a$.

#### Proposition 5.1

The random dynamical system (2.3) has the following properties:

- if $\lambda =0$, then the two-point motion admits an infinite stationary Radon measure $\mu ^{(2)}$ on $\mathbb {T}^2 \setminus \Delta$, and
- if $\lambda >0$, then the two-point motion admits a stationary probability measure $\mu ^{(2)}$ on $\mathbb {T}^2 \setminus \Delta$.

#### Proof

For a fixed small $\varepsilon >0$ take the compact set

$$\begin{aligned} \mathcal {K}&= \mathbb {T}^2 \setminus \Delta _\varepsilon . \end{aligned}$$

Because *T* is of degree two, each element in $\mathbb {T}$ has two distinct pre-images. The minimal distance between pre-images is smaller than $R_{\min }$ (recall (2.1)). Take $\varepsilon < R_{\min }$, so that points in $\mathbb {T}^2 \setminus (\mathcal {K} \cup \Delta )$ can not be mapped into $\Delta$ by $T^{(2)}_a$. Note that there will be points in $\mathcal {K}$ that are mapped into $\Delta$ by some $T^{(2)}_a$. Namely, any $(x,y) \in \mathbb {T}^2$ with $x\ne y$ and $T_a (x) = T_a(y)$ lies in $\mathcal {K}$ and will be mapped into $\Delta$ by $T^{(2)}_a$. By Lemma 4.2, for any $(x_0,y_0)\in \mathcal {K}$, of the random orbit $(x_n,y_n) = \left( T^{(2)}_\omega \right) ^n(x_0,y_0)$ has almost surely infinitely many points contained in $\mathcal {K}$.

Fix a smooth function $\xi : \mathbb {T}^2 \rightarrow [0,1]$ with support disjoint from $\Delta$ and with $\xi \equiv 1$ on $\mathcal {K}$. For an initial point $(x_0,y_0)$ consider the random stopping time $V(\omega ) \ge 1$ (suppressing dependence on $(x_0,y_0)$) so that the probability $\mathbb {P} (V = n+1 \; | \; V \ge n)$ equals $\xi (x_{n+1},y_{n+1})$. So when the random orbit arrives at $(x_{n+1},y_{n+1})$ we stop with probability $\xi (x_{n+1},y_{n+1})$ and continue with probability $1 - \xi (x_{n+1},y_{n+1})$.

We use $\mathbb {E}$ to denote the expectation both over ${\Sigma _\vartheta }$ and over the random process defining the random stopping time. So $\mathbb {E} \left[ \delta _{(x_V,y_V)} \right]$ is the distribution of the stopped point $(x_V,y_V)$. It is an element of the space $\mathcal {P} (\textrm{supp}\, \xi )$ of probability measures on $\textrm{supp}\, \xi$, which we endow with the weak star topology.

We claim that this distribution depends continuously on $(x_0,y_0)\in \mathcal {K}$ in the weak star topology. Namely, consider a sequence of distributions of stopped points for a converging sequence of initial points $(x_0^n,y_0^n)$. From (4.13) we get that for $\zeta >0$ small there exists $N>0$ so that with probability at least $1 - \zeta$, the stopping time *V* is smaller than *N*. As the maps $T_a$ depend continuously on *a*, the points $(x_V,y_V)$, $V<N$, for $(x_0^n,y_0^n)$ are close to those for $(x_0,y_0)$. Consequently, the distribution $\mathbb {E} \left[ \delta _{(x_V,y_V )} \right]$ of the stopped point depends continuously on $(x_0,y_0)$, for $(x_0,y_0) \in \mathcal {K}$, in the weak star topology.

Define

5.1 $$\begin{aligned} P_\xi \mu&= \int _{\mathbb {T}^2} \mathbb {E} \left[ \delta _{(x_V,y_V)} \right] \, d\mu (x_0,y_0) \end{aligned}$$

acting on $\mathcal {P} (\textrm{supp}\, \xi )$. Then (5.1) is a continuous map from $\mathcal {P} (\textrm{supp}\, \xi )$ to itself. We can therefore apply the Krylov-Bogolyubov procedure of taking a converging subsequence of Césaro averages, to find a $P_\xi$ invariant probability measure $\varsigma _0$,

$$\begin{aligned} P_\xi \varsigma _0 = \varsigma _0, \end{aligned}$$

with $${ \text {supp}\,} \varsigma _0 \subset \text {supp}\, \xi $$.

We will use this to construct a stationary Radon measure on $\mathbb {T}^2 \setminus \Delta$. Consider the average measures

5.2 $$\begin{aligned} \overline{m}_{(x_0,y_0)}&:=\mathbb {E} \left[ \sum _{j=0}^{V(\omega )-1} \delta _{(x_j,y_j)} \right] . \end{aligned}$$

Because $1 - \xi$ vanishes on $\mathcal {K}$, we can write, with $(x_j,y_j) = T^{(2)}_{\omega _{j-1}} \circ \cdots \circ T^{(2)}_{\omega _0} (x_0,y_0)$,

5.3 $$\begin{aligned} \overline{m}_{(x_0,y_0)}&= \sum _{n=0}^\infty \,\,\,\iint \displaylimits _{\overset{\omega _0,\ldots ,\omega _{n-1}}{\in \mathbb {T}}}\, \prod _{j=1}^n \left( 1 - \xi (x_j,y_j) \right) \delta _{(x_n,y_n)} \, d\mathbb {P}(\omega _0) \cdots d\mathbb {P}(\omega _{n-1}). \end{aligned}$$

Integrate over $(x_0,y_0)$ taken from the measure $\varsigma _0$ to obtain

5.4 $$\begin{aligned} \mu ^{(2)}&:=\int _{\mathbb {T}^2} \overline{m}_{(x_0,y_0)} \, d\varsigma _0 (x_0,y_0). \end{aligned}$$

Written out this reads

$$\begin{aligned} \mu ^{(2)}(A)&= \int _{\mathbb {T}^2} \mathbb {E} \left[ \sum _{j=0}^{V(\omega )-1} 1\!\!1_A(T^j_\omega (x),T^j_\omega (y))\right] \, d\varsigma _0 (x_0,y_0) \end{aligned}$$

for Borel sets $A \subset \mathbb {T}^2 \setminus \Delta$.

We claim that for any continuous function $\psi : \mathbb {T}^2 \rightarrow \mathbb {R}$ with support disjoint from $\Delta$,

5.5 $$\begin{aligned} \int _{\mathbb {T}^2} \psi \, d m&= \int _{\mathbb {T}^2} \mathbb {E} \left[ \sum _{j=0}^{V(\omega )-1} \psi (x_j)\right] \, d\varsigma _0 (x_0,y_0) \end{aligned}$$

is well defined and finite. We conclude from this that $\mu ^{(2)}$ is a Radon measure that assigns finite measure to compact sets disjoint from $\Delta$. To establish the claim note that there are $N>0$ and $q >0$ so that with probability at least *q* an orbit starting in $\text {supp}\, \psi$ hits $\mathcal {K}$ in at most *N* steps. Note that iterates outside $\text {supp}\, \psi$ do not contribute to the right hand side of (5.5). We find an estimate

$$\begin{aligned} \mathbb {E} \left[ \sum _{j=0}^{V(\omega )-1} \psi (x_j,y_j) \right]< \sum _{i=1}^\infty \max _{(x,y)\in \mathbb {T}^2} |\psi (x,y)| N (1-q)^{i-1} < \infty . \end{aligned}$$

That is, $\mathbb {E} \left[ \sum _{j=0}^{V(\omega )-1} |\psi (x_j,y_j)| \right]$ is finite and bounded uniformly on $\text {supp}\,\psi$. This implies that the right hand side of (5.5) is finite.

We will establish that $\mu ^{(2)}$ is stationary for the two-point maps. We must thus show $P \mu ^{(2)} = \mu ^{(2)}$ with

$$\begin{aligned} P \mu ^{(2)} :=\int _{\mathbb {T}} \left( T_a^{(2)}\right) _*\mu ^{(2)} \, d\mathbb {P} (a). \end{aligned}$$

Applying *P* yields, with $(x_j,y_j) = T^{(2)}_{\omega _{j-1}} \circ \cdots \circ T^{(2)}_{\omega _0} (x_0,y_0)$,

$$\begin{aligned} P \overline{m}_{x_0,y_0}&= \int _{\mathbb {T}} \left( T^{(2)}_a \right) _*\overline{m}_{x_0,y_0} \, d\mathbb {P} (a) \\&= \sum _{n=0}^\infty \,\,\, \iint \displaylimits _{\overset{a,\omega _0,\ldots ,\omega _{n-1}}{ \in \mathbb {T}}} \, \prod _{j=1}^n \left( 1 - \xi ( x_j,y_j) \right) \left( T^{(2)}_a \right) _*\delta _{(x_n,y_n)} \, d\mathbb {P}(a) d\mathbb {P}(\omega _0) \cdots d\mathbb {P}(\omega _{n-1}) \\&= \sum _{n=0}^\infty \,\,\,\iint \displaylimits _{\overset{\omega _0,\ldots ,\omega _{n}}{\in \mathbb {T}}} \, \prod _{j=1}^n \left( 1 - \xi (x_j,y_j) \right) \delta _{(x_{n+1},y_{n+1})} \, d\mathbb {P}(\omega _0) d\mathbb {P}(\omega _1) \cdots d\mathbb {P}(\omega _{n}) \\&= \mathbb {E} \left[ \sum _{j=1}^{V(\omega )} \delta _{(x_j,y_j)} \right] \end{aligned}$$

(again using that $1-\xi$ vanishes on $\mathcal {K}$).

Compared to (5.2), the index *j* is counting from 1 to $V(\omega )$ instead of from 0 to $V(\omega )-1$. We thus find

$$\begin{aligned} P \overline{m}_{(x_0,y_0)}&= \overline{m}_{(x_0,y_0)} - \delta _{(x_0,y_0)} + \mathbb {E} \left[ \delta _{(x_{V(\omega )},y_{V(\omega )})}\right] . \end{aligned}$$

Integration over $\varsigma _0$ yields

$$\begin{aligned} P \mu ^{(2)}&= P \int _{\mathbb {T}^2} \overline{m}_{(x_0,y_0)} \, d\varsigma _0 (x_0,y_0) \\&= \int _{\mathbb {T}^2} P \overline{m}_{(x_0,y_0)} \, d\varsigma _0 (x_0,y_0) \\&= \int _{\mathbb {T}^2} \overline{m}_{(x_0,y_0)} \, d\varsigma _0 (x_0,y_0) - \int _{\mathbb {T}^2} \delta _{(x_0,y_0)} \, d\varsigma _0 (x_0,y_0) \\&\qquad + \int _{\mathbb {T}^2} \mathbb {E} \left[ \delta _{(x_{V(\omega )},y_{V(\omega )})} \right] \, d\varsigma _0 (x_0,y_0) \\&= \mu ^{(2)} - \varsigma _0 + P_\xi \varsigma _0 \\&= \mu ^{(2)}, \end{aligned}$$

the last step by $P_\xi$ invariance of $\varsigma _0$.

Note that the argument includes the observation that $\mu ^{(2)}$ assigns finite measure to compact sets disjoint from $\Delta$.

For $\lambda = 0$, Lemma 2.5 shows that iterates of points $(x,y) \in \mathbb {T}^2$ enter any small neighborhood of $\Delta$ with positive probability. Combining this with Lemma 4.2 shows

$$\begin{aligned} \mu ^{(2)}(\mathbb {T}^2)&= \int _{\mathbb {T}^2} \mathbb {E} \left[ \sum _{j=0}^{V(\omega )-1} 1\!\!1_{\mathbb {T}^2}(T^j_\omega (x),T^j_\omega (y))\right] \, d\varsigma _0 (x_0,y_0)\\&= \infty . \end{aligned}$$

For $\lambda >0$, by Lemma 4.4, we see that

$$\begin{aligned} X= &   \int _{\mathbb {T}^2} \mathbb {E} \left[ \sum _{j=0}^{V(x,y,\omega )-1} 1\!\!1_{\mathbb {T}^2}(T^j_\omega (x),T^j_\omega (y))\right] \, d\varsigma _0 (x,y)\\= &   \int _{\mathbb {T}^2} \mathbb {E} \left[ V(x,y,\omega ) \right] \,d\varsigma _0 (x,y)<\infty . \end{aligned}$$

Now $\mu ^{(2)}$ given by

$$\begin{aligned} \mu ^{(2)}(A)&= \frac{1}{X}\int _{\mathbb {T}^2} \mathbb {E} \left[ \sum _{j=0}^{V(x,y,\omega )-1} 1\!\!1_{A}(T^j_\omega (x),T^j_\omega (y))\right] \, d\varsigma _0(x,y) \end{aligned}$$

is a stationary measure. Since $\mu ^{(2)} (\mathbb {T}^2) = 1$, we find from this expression that $\mu ^{(2)}$ is a probability measure. $\square$

#### Remark 5.2

The stationary measure $\mu ^{(2)}$ is obtained by pushing forward the measure $\varsigma _0$ on the support of a test function $\xi$. The test function is constant one on a suitable set $\mathcal {K}$. The construction shows that $\mu ^{(2)}$ restricted to $\mathcal {K}$ equals $\varsigma _0$ restricted to $\mathcal {K}$ (see (5.3) and (5.4)). We can therefore also obtain $\mu ^{(2)}$ from $\varsigma _0$ restricted to $\mathcal {K}$ by pushing forward. That is, with

$$\begin{aligned} \widehat{m}_{(x_0,y_0)}&= \sum _{n=0}^\infty \,\,\,\iint \displaylimits _{\overset{\omega _0,\ldots ,\omega _{n-1}}{\in \mathbb {T}}}\, \prod _{j=1}^n \left( 1 - 1\!\!1_\mathcal {K} (x_j,y_j) \right) \delta _{(x_n,y_n)} \, d\lambda (\omega _0) \cdots d\lambda (\omega _{n-1}) \end{aligned}$$

for $(x_0,y_0) \in \mathcal {K}$, we find

$$\begin{aligned} \mu ^{(2)}&= \int _{\mathcal {K}} \widehat{m}_{(x_0,y_0)} \, d\varsigma _0 (x_0,y_0). \end{aligned}$$

$$\square $$

### The support of the stationary measure

Hypothesis (H5) and Lemma 2.4 show that for all $\varepsilon >0$, there exists a $k\in \mathbb {N}$, such that images under $\Theta ^{(2)}$ of a set $\Sigma _\vartheta \times \{(x,y)\}$ for $(x,y) \in \mathbb {T}^2 {\setminus } \Delta _\varepsilon$, cover $\Sigma _\vartheta \times \mathbb {T}^2$. This implies that stationary measures for the two-point motion have full support, if they are obtained from an inducing scheme as in Sect. 5.1.

### The growth rate of the stationary measure at the diagonal for $\lambda =0$

The following lemma discusses the expected number of such orbit points that lie inside strips $\Delta _\delta {\setminus } \Delta _\varepsilon$, in the limit of $\varepsilon$ going to zero. The obtained bounds will be used below to derive the growth-rate near the diagonal of stationary measures for the two-point motion.

#### Lemma 5.3

Let *R*, *K* be as in Lemma 4.1. Suppose $\lambda = 0$. Assume $0<\varepsilon<\delta <R$. For $(x,y) \in \mathbb {T}^2$ with $0< d(x,y) < \delta$, define

$$\begin{aligned} g_{\varepsilon ,\delta }(x,y) = \mathbb {E} \left[ \sum _{i=0}^{\tau _{\delta ,+}(x,y)} 1\!\!1_{[\varepsilon ,\infty ) }(d( T_\omega ^i (x),T_\omega ^i (y))) \right] . \end{aligned}$$

Then

$$\begin{aligned}  &   \frac{1}{V}\left( \ln \left( \frac{\delta a_1}{d(x,y)}\right) -6K \right) \le \liminf _{\varepsilon \rightarrow 0} \frac{g_{\varepsilon ,\delta }(x,y)}{-\ln (\varepsilon )}\\  &   \quad \le \limsup _{\varepsilon \rightarrow 0} \frac{g_{\varepsilon ,\delta }(x,y)}{-\ln (\varepsilon )} \le \frac{1}{V}\left( \ln \left( \frac{\delta a_2}{d(x,y)}\right) + 6K \right) . \end{aligned}$$

#### Proof

We follow [11, Proposition 5.6], with modifications needed for the discrete time setting. Recall that $a_1,a_2$ are given in Hypothesis (H1). Define, for $\varepsilon<r<\delta$,

$$\begin{aligned} g_{\varepsilon ,\delta }^-(r)&= \inf \left\{ g_{\varepsilon ,\delta }(x,y) \; ; \; ra_1\le d(x,y) \le r \right\} , \\ g_{\varepsilon ,\delta }^+(r)&= \sup \left\{ g_{\varepsilon ,\delta }(x,y) \; ; \; r \le d(x,y) \le r a_2 \right\} . \end{aligned}$$

Observe that $r \mapsto g^\pm _{\varepsilon ,\delta }(r)$ is a monotone non-increasing function on $[\varepsilon ,\delta ]$, and constant on $(0,\varepsilon ]$.

We first focus on $g_{\varepsilon ,\delta }^-$. Conditioning on the smallest stopping time $\tau _{\delta ,+}$ or $\tau _{\varepsilon ,-}$, which have been defined in (4.14.2) and (4.14.2), we obtain

$$\begin{aligned} g_{\varepsilon ,\delta }(x,y)&= \mathbb {P}\left( \tau _{\varepsilon ,-}(x,y)> \tau _{\delta ,+}(x,y)\right) \mathbb {E} [\tau _{\delta ,+}(x,y)] \\&\qquad + \mathbb {P}\left( \tau _{\varepsilon ,-}(x,y)< \tau _{\delta ,-}(x,y)\right) \mathbb {E} [\tau _{\varepsilon ,-}(x,y)] \\&\qquad + \mathbb {P}\left( \tau _{\varepsilon ,-}(x,y)< \tau _{\delta ,-}(x,y)\right) \mathbb {E} \left[ g_{\varepsilon ,\delta }(x_{\tau _{\varepsilon ,-}},y_{\tau _{\varepsilon ,-}})\mid \tau _{\varepsilon ,-}(x,y)< \tau _{\delta ,+}(x,y) \right] \\&= \mathbb {E} \left[ \min \{\tau _{\delta ,+}(x,y),\tau _{\varepsilon ,-}(x,y)\}\right] \\&\qquad + \mathbb {P}\left( \tau _{\varepsilon ,-}(x,y)< \tau _{\delta ,+}(x,y)\right) \mathbb {E} \left[ g_{\varepsilon ,\delta }(x_{\tau _{\varepsilon ,-}},y_{\tau _{\varepsilon ,-}})\mid \tau _{\varepsilon ,-}(x,y)< \tau _{\delta ,+}(x,y) \right] \\&\ge \mathbb {E} \left[ \min \{\tau _{\delta ,+}(x,y),\tau _{\varepsilon ,-}(x,y)\}\right] + g^-_{\varepsilon ,\delta }(\varepsilon )\mathbb {P}\left( \tau _{\varepsilon ,-}(x,y)< \tau _{\delta ,+}(x,y)\right) . \end{aligned}$$

Due to monotonicity of $g_{\varepsilon ,\delta }^-(r)$ in *r*, we have for $\varepsilon< r < \delta$,

$$\begin{aligned} g_{\varepsilon ,\delta }^-(\varepsilon )&\ge g_{\varepsilon ,\delta }^-(r)\\&\ge \inf \left\{ g_{\varepsilon ,\delta }(x,y) \; ; \; a_1 r \le d(x,y) \le r \right\} \\&\ge \inf _{ra_1\le d(x,y) \le r}\left\{ \mathbb {E} \left[ \min \{\tau _{\delta ,+}(x,y),\tau _{\varepsilon ,-}(x,y)\}\right] + g_{\varepsilon ,\delta }^-(\varepsilon ) \mathbb {P}\left( \tau _{\varepsilon ,-}(x,y)< \tau _{\delta ,+}(x,y)\right) \right\} . \end{aligned}$$

We get the lower bound for $g_{\varepsilon ,\delta }(x,y)$ by setting $r = d(x,y)$, using (4.4) and (4.5) from Lemma 4.1, and $g_{\varepsilon ,\delta }^-(\varepsilon )\ge g_{\varepsilon ,\delta }^-(r)$,

$$\begin{aligned}  &   g_{\varepsilon ,\delta }(x,y)\ge g_{\varepsilon ,\delta }^-(r) \\  &   \quad \ge \inf _{ra_1\le d(x,y)\le r}\Bigg \{\frac{1}{V}\left( \ln \left( \frac{\delta }{d(x,y)} \right) \ln \left( \frac{d(x,y)}{\varepsilon } \right) - 6K |\ln \varepsilon | + 2K^2 \right) \\  &   \qquad + g_{\varepsilon ,\delta }^-(\varepsilon ) \frac{\displaystyle \ln \left( \frac{\delta }{d(x,y)}\right) -2K}{\displaystyle \ln \left( \frac{\delta }{\varepsilon }\right) }\Bigg \} \\  &   \quad \ge \frac{1}{V} \inf _{r/a\le d(x,y)\le r}\Bigg \{ \ln \left( \frac{\delta }{d(x,y)} \right) \ln \left( \frac{d(x,y)}{\varepsilon } \right) \Bigg \}\\  &   \quad - 6K |\ln (\varepsilon )| + 2K^2 + g_{\varepsilon ,\delta }^-(r) \frac{\displaystyle \ln \left( \frac{\delta }{ra_1}\right) -2K}{\displaystyle \ln \left( \frac{\delta }{\varepsilon }\right) }. \end{aligned}$$

Divide by $|\ln (\varepsilon )|$ and take $\liminf _{\varepsilon \rightarrow 0}$. This yields

$$\begin{aligned} \liminf _{\varepsilon \rightarrow 0}\frac{g_{\varepsilon ,\delta }^-(\delta )}{|\ln (\varepsilon )|}\ge \inf _{a_1 r<d(x,y)<r} \frac{\ln \left( \frac{\delta }{d(x,y)} \right) - 6K }{V} \\ \ge \frac{\ln \left( \frac{\delta }{r/a} \right) - 6K }{V}. \end{aligned}$$

The bound for $g^+_{\varepsilon ,\delta } (r)$ is obtained by following a similar scheme. $\square$

The following proposition allows us to estimate on the growth-rate of the stationary measure $\mu ^{(2)}$ near the diagonal, in the case $\lambda = 0$.

#### Proposition 5.4

Suppose $\lambda = 0$. Given the stationary measure $\mu ^{(2)}$, there exist $\alpha ,\beta \in (0,\infty )$, such that

5.6 $$\begin{aligned} \alpha \le \liminf _{\varepsilon \rightarrow 0} \frac{\mu ^{(2)}(\mathbb {T}^2\setminus \Delta _\varepsilon )}{-\ln (\varepsilon )} \le \limsup _{\varepsilon \rightarrow 0} \frac{\mu ^{(2)}(\mathbb {T}^2\setminus \Delta _\varepsilon )}{-\ln (\varepsilon )} \le \beta . \end{aligned}$$

#### Proof

Temporary fix $\varepsilon >0$ small. For $0< \varepsilon < \delta$ for suitable small $\delta$, set $\mathcal K = \mathbb T^2 {\setminus } \Delta _\delta$. For $(x,y) \in \mathcal {K}$, write

$$\begin{aligned} N (x,y,\omega ) = \min \{ n > 0 \;; \; (T^n_\omega (x), T^n_\omega (y)) \in \mathcal {K} \} \end{aligned}$$

for the first return time to $\mathcal {K}$. By Remark 5.2 we have that for all stationary Radon measures $\mu ^{(2)}$ on $\mathbb {T}^2 \setminus \Delta$, the restricted measure $\mu _\mathcal {K} = \mu ^{(2)}\vert _\mathcal {K}$ is a stationary measure for the induced process

$$\begin{aligned} (x_{n+1},y_{n+1}) = \left( T_\omega ^{(2)}\right) ^{N(x_n,y_n,\omega )} (x_n,y_n) \end{aligned}$$

on $\mathcal K$. For convenience we rescale $\mu _{\mathcal {K}}$ so that it becomes a probability measure,

$$\begin{aligned} \mu _{\mathcal {K}} (\mathcal {K}) = 1. \end{aligned}$$

Denote the set $\mathcal {G} \subset \mathcal {K}\times {\Sigma _\vartheta }$ as the union of $(x,y,\omega ) \in \mathcal {K}\times {\Sigma _\vartheta }$, such that there exists $\tau (x,y,\omega )\in \mathbb {N}$ with the following properties,

1. for $$0< i <\tau $$, $$(T_\omega ^i(x),T_\omega ^i(y)) \notin \mathcal {K}$$,
2. $$(T_\omega ^{\tau (x,y,\omega )}(x),T_\omega ^{\tau (x,y,\omega )}(y)) \in \Delta _{e^{-7K} a_1 \delta }$$.

Here *K* is as in Lemma 4.1. By Lemma 2.5, we have $(\mu _\mathcal {K} \times \mathbb P) (\mathcal G) > 0$.

Now we have the ingredients to prove the lower bound for the $\liminf$. For all measurable sets $A \subset \mathbb T^2$, from Remark 5.2 we obtain

5.7 $$\begin{aligned} \mu ^{(2)}(A) = \int _{\mathcal K \times {\Sigma _\vartheta }} \sum _{j=0}^{N(x,y,\omega )-1} 1\!\!1_{A}(T^j_\omega (x),T^j_\omega (y)) \, d(\mu _\mathcal {K}\times \mathbb P) (x,y,\omega ). \end{aligned}$$

By (5.7) and Lemma 5.3,

$$\begin{aligned} \mu ^{(2)}(\mathbb T^2 \setminus \Delta _{\varepsilon })&= \int _{\mathcal K \times {\Sigma _\vartheta }} \sum _{j=0}^{N(x,y,\omega )-1} 1\!\!1_{\mathbb T^2 \setminus \Delta _{\varepsilon }}(T^j_\omega (x),T^j_\omega (y)) \, d(\mu _\mathcal {K}\times \mathbb P) (x,y,\omega )\\&\ge \int _\mathcal {G} \sum _{j=0}^{N(x,y,\omega )-1} 1\!\!1_{\mathbb T^2 \setminus \Delta _{\varepsilon }}(T^j_\omega (x),T^j_\omega (y)) \, d(\mu _\mathcal {K}\times \mathbb P) (x,y,\omega )\\&\ge \int _\mathcal {G} \sum _{j=\tau (x,y,\omega )}^{N(x,y,\omega )-1} 1\!\!1_{\mathbb T^2 \setminus \Delta _{\varepsilon }}(T^j_\omega (x),T^j_\omega (y)) \, d(\mu _\mathcal {K}\times \mathbb P) (x,y,\omega )\\&\ge \int _\mathcal {G} g_{\varepsilon ,\delta }\left( \left( T_\omega ^{(2)}\right) ^{\tau (x,y,\omega )}(x,y)\right) \, d(\mu _\mathcal {K}\times \mathbb P) (x,y,\omega ). \end{aligned}$$

To get the first inequality of (5.6) we divide both sides by $-\ln (\varepsilon )$, take the $\liminf$, and apply Lemma 5.3. This yields

$$\begin{aligned} \liminf _{\varepsilon \rightarrow 0}\frac{\mu ^{(2)}(\mathbb T^2 \setminus \Delta _{\varepsilon })}{-\ln (\varepsilon )}&\ge \frac{(K + \ln (a_1)) (\mu _K\times \mathbb P) (\mathcal G)}{V}. \end{aligned}$$

This proves the lower bound for the $\liminf$.

To get the upper bound for the $\limsup$ we use a similar technique. Here we have to incorporate the possibility that points in $\mathcal {K}$ are mapped onto, or close to, $\Delta$ by a single iterate of $T^{(2)}_\omega$. When pushing forward $\mu _{\mathcal {K}}$, this moves mass directly to small neighborhoods of $\Delta$. Write $\mathcal {K} \times \Sigma _\vartheta = \mathcal {R}_0 \cup \mathcal {R}_+$ as a union of sets on which *N* is either equal to 1 or is bigger than 1,

$$\begin{aligned} \mathcal {R}_0 = \left\{ (x,y,\omega ) \in \mathcal {K} \times \Sigma _\vartheta \; ; \; N(x,y,\omega ) = 1\right\} , \\ \mathcal {R}_+ = \left\{ (x,y,\omega ) \in \mathcal {K} \times \Sigma _\vartheta \; ; \; N(x,y,\omega ) > 1\right\} . \end{aligned}$$

The set $\mathcal {R}_+$ is a disjoint union of a set

$$\begin{aligned} \mathcal {G}_d = \mathcal {R}_+ \cap \left( \Delta _{R_\text {min}} \times \Sigma _\vartheta \right) \end{aligned}$$

(recall (2.1) for the definition of $R_{\min }$) and its complement $\mathcal {G}_c$, which is contained in $\left( \Delta _{a_2 \delta }{\setminus } \Delta _\delta \right) \times \Sigma _\vartheta$. For $(x,y,\omega ) \in \mathcal {G}_c$ we find $T^{(2)}_\omega (x,y) \subset \Delta _{\delta }{\setminus } \Delta _{a_1 \delta }$. We have

5.8 $$\begin{aligned}  &   \mu ^{(2)}(\mathbb T^2 \setminus \Delta _{\varepsilon }) = \int _\mathcal {K} \int _{{\Sigma _\vartheta }} \sum _{j=0}^{N(x,y,\omega )-1} 1\!\!1_{\mathbb T^2 \setminus \Delta _{\varepsilon }}(T^j_\omega (x),T^j_\omega (y)) \, d\mathbb P(\omega )d\mu _\mathcal {K} (x,y) \nonumber \\  &   \quad = \int _\mathcal {K} \int _{{\Sigma _\vartheta }} \sum _{j=0}^{N(x,y,\omega )-1} 1\!\!1_{\mathbb T^2 \setminus \Delta _{\varepsilon }}(T^j_\omega (x),T^j_\omega (y)) \, d\mathbb P(\omega )d\mu _\mathcal {K} (x,y) \nonumber \\  &   \quad \le \iint _{\mathcal {R}_0}\, d\mathbb P(\omega )d\mu _\mathcal {K} (x,y) + \iint _{\mathcal {R}_+} \sum _{j=1}^{N(x,y,\omega )-1} 1\!\!1_{\mathbb T^2 \setminus \Delta _{\varepsilon }}(T^j_\omega (x),T^j_\omega (y)) \, d\mathbb P(\omega )d\mu _\mathcal {K} (x,y) \nonumber \\  &   \quad \le \mu _\mathcal {K} (\mathcal {K}) + \iint _{\mathcal {R}_+} g_{\varepsilon ,\delta }( T_\omega ^{(2)}(x,y) ) \, d\mathbb P(\omega )d\mu _\mathcal {K} (x,y) . \end{aligned}$$

Recall that by Lemma 2.3, we have the existence of $C_1>0$ with

5.9 $$\begin{aligned} \mathbb {E} \left[ -\ln (d(T_\omega ^{(2)}(x,y))) \right] < C_1, \end{aligned}$$

for all $$(x,y) \in \mathcal {K}$$.

To conclude the proposition we divide (5.8) by $-\ln (\varepsilon )$ and take the $\limsup$. Doing this, applying Fatou’s lemma and using Lemma 5.3 and (2.4), yields

$$\begin{aligned}  &   \limsup _{\varepsilon \rightarrow 0} \frac{\mu ^{(2)}(\mathbb T^2 \setminus \Delta _{\varepsilon })}{-\ln (\varepsilon )} \\  &   \quad \le \limsup _{\varepsilon \rightarrow 0} \frac{\mu _\mathcal {K} (\mathcal {K})}{-\ln (\varepsilon )}+ \limsup _{\varepsilon \rightarrow 0} \iint _{\mathcal {G}_c \cup \mathcal {G}_d} \frac{g_{\varepsilon ,\delta }(T_\omega ^{(2)}(x,y) )}{-\ln (\varepsilon )} \, d\mathbb P(\omega )d\mu _\mathcal {K} (x,y) \\  &   \quad \le \iint _{\mathcal {G}_c \cup \mathcal {G}_d} \limsup _{\varepsilon \rightarrow 0}\frac{g_{\varepsilon ,\delta }(T_\omega ^{(2)}(x,y) )}{-\ln (\varepsilon )} \, d\mathbb P(\omega )d\mu _\mathcal {K} (x,y) \\  &   \quad \le \iint _{\mathcal {G}_c \cup \mathcal {G}_d} \frac{1}{V}\left( \ln \left( \frac{\delta a_2}{d(T_\omega ^{(2)}(x,y) )}\right) + 6K \right) \, d\mathbb P(\omega )d\mu _\mathcal {K} (x,y) \\  &   \quad \le \frac{\ln (\delta a_2) + 6K + C_1}{V}, \end{aligned}$$

which finishes the proof. $\square$

### The growth rate of the stationary measure at the diagonal for $\lambda >0$

The following proposition allows us to estimate on the growth-rate of the stationary measure $\mu ^{(2)}$ near the diagonal, in the case of $\lambda >0$.

#### Proposition 5.5

Suppose $\lambda >0$ and let $\gamma$ be the negative value so that $\Lambda (\gamma )=0$. Suppose $\gamma \in (-1/2,0)$. Given the stationary measure $\mu ^{(2)}$, there exist $\alpha ,\beta \in (0,\infty )$, such that

5.10 $$\begin{aligned} \alpha \le \liminf _{\varepsilon \rightarrow 0} \frac{\mu ^{(2)}(\Delta _\varepsilon )}{\varepsilon ^{-\gamma }} \le \limsup _{\varepsilon \rightarrow 0} \frac{\mu ^{(2)}(\Delta _\varepsilon )}{\varepsilon ^{-\gamma }} \le \beta . \end{aligned}$$

To prove the above proposition, we first consider orbits near the diagonal.

#### Lemma 5.6

Let *R* be as in Lemma 4.1. Suppose $\lambda > 0$. Assume $0< \varepsilon<\delta <R$. For $(x,y) \in \mathbb {T}^2$ with $0< d(x,y) < \delta$, define

$$\begin{aligned} f_{\varepsilon ,\delta } (x,y) = \mathbb {E}\left[ \sum _{i=0}^{\tau _{\delta ,+}(x,y,\omega )-1}1\!\!1_{(0,\varepsilon ]}(d(T_\omega ^i(x),T_\omega ^i(y))) \right] . \end{aligned}$$

There exist $K < \infty$, $\kappa \in (0,1)$, such that for $(x,y) \in \mathbb T^2$, if $0<\varepsilon<d(x,y)<\kappa \delta <\kappa R$, then

$$\begin{aligned} \frac{1}{K}\left( \frac{d(x,y)}{\varepsilon }\right) ^\gamma \le f_{\varepsilon ,\delta } (x,y) \le {K}\left( \frac{d(x,y)}{\varepsilon }\right) ^\gamma . \end{aligned}$$

#### Proof

Let $\kappa$ be as in Lemma 4.5. Let $K_0$ be *K* from Lemma 4.5 and set $K_1 = e^{2 \lambda K_0}$. By a straightforward computation for the lower bound, comparable to the proof of Lemma 5.3, we obtain

$$\begin{aligned} f_{\varepsilon ,\delta }(x,y)&\ge \mathbb {P}\left( \tau _{\varepsilon /K_1,-}(x,y)< \tau _{\delta ,+}(x,y)\right) \\&\qquad \times \mathbb {E}\left[ \tau _{\varepsilon ,+}(T^{(2)\tau _{\varepsilon /K_1}}_\omega (x,y)) \mid \tau _{\varepsilon /K_1,-}(x,y)< \tau _{\delta ,+}(x,y) \right] \\&\ge \frac{1}{K_0}\left( \frac{K_1 d(x,y)a_1}{\varepsilon }\right) ^\gamma \left( \frac{1}{\lambda }\left( \ln \left( \frac{a_1}{K_1 }\right) -2K_0\right) \right) . \end{aligned}$$

Set

5.11 $$\begin{aligned} f_{\varepsilon ,\delta }^+(r) = \sup _{r a_1 < d(x,y)\le r} \mathbb {E}\left[ \sum _{i=0}^{\tau _{\delta ,+}(x,y,\omega )-1} 1\!\!1_{(0,\varepsilon ]}(d(T^{i}_\omega (x),T^i_\omega (y)))\right] \end{aligned}$$

and observe that $f_{\varepsilon ,\delta }^+$ is monotone decreasing in *r*. Let $K_2 = \left( \frac{1}{2K_0} \right) ^{1/\gamma }/a_2$. When $K_2\varepsilon < \kappa R$, we have

$$\begin{aligned} f_{\varepsilon ,\delta }^+(\varepsilon )&\le \sup _{\varepsilon a_1< d(x,y)\le \varepsilon } \mathbb {E}\left[ \tau _{(K_2\varepsilon ), +}(x,y)\right] + \sup _{\varepsilon K_2< d(x,y)\le \varepsilon K_2 a_2} \mathbb {P}\left( \tau _{\varepsilon ,-}(x,y)< \tau _{\delta ,+}(x,y)\right) f_{\varepsilon ,\delta }^+(\varepsilon )\\&\le \frac{1}{\lambda } \left( \ln \left( \frac{K_2}{a_1}\right) + 2K_0 \right) + K_0\left( {K_2a_2} \right) ^\gamma f_{\varepsilon ,\delta }^+(\varepsilon ). \end{aligned}$$

Therefore $f_{\varepsilon ,\delta }^+(\varepsilon ) < \infty$. So, using $f_{\varepsilon ,\delta }(x,y) \le \mathbb {P}\left( \tau _{\varepsilon ,-}(x,y)< \tau _{\delta ,+}(x,y)\right) f_{\varepsilon ,\delta }^+(\varepsilon )$, we get the desired result. $\square$

#### Proof of Proposition 5.5

We follow reasoning of Proposition 5.4, using Lemmas 4.4 and 5.6. Let $R,K>0$ be as in Lemma 5.6. For $0<\delta <R$ small, take $\mathcal {K} = \mathbb {T}^2 \setminus \Delta _\delta$. As in Proposition 5.1, a finite measure $\sigma _0$ is constructed and from the restriction $\mu _\mathcal {K}$ of $\sigma _0$ to $\mathcal {K}$, the measure $\mu ^{(2)}$ is obtained by pushing forward $\mu _\mathcal {K}$ by the two-point maps. See Remark 5.2.

By rescaling $\mu _\mathcal {K}$ we may assume

$$\begin{aligned} \int _{\mathbb {T}^2} \mathbb {E} \left[ V(x,y,\omega ) \right] \, d\mu _\mathcal {K} (x,y)=1. \end{aligned}$$

Fix $\varepsilon > 0$ small enough and let $\delta <R$. For the stationary measure of the two-point motion we have by Proposition 5.1 that

$$\begin{aligned} \mu ^{(2)}(\Delta _\varepsilon )&= \int _{\mathbb {T}^2} \mathbb {E} \left[ \sum _{j=0}^{V(x,y,\omega )-1} 1\!\!1_{\Delta _\varepsilon }(T^j_\omega (x),T^j_\omega (y))\right] \, d\mu _\mathcal {K} (x_0,y_0). \end{aligned}$$

We remark that the condition $|\gamma |<1/2$ is to bound the mass that is transported to neighborhoods of the diagonal from outside $\Delta _{R_\text {min}}$ by this construction (recall (2.1) for the definition of $R_{\min }$).

As before, see the proof of Proposition 5.4, denote the set of $\mathcal {G} \subset (\mathbb {T}^2{\setminus } \Delta _\delta ) \times {\Sigma _\vartheta }$ as the union of $(x,y,\omega ) \in (\mathbb {T}^2{\setminus } \Delta _\delta )\times {\Sigma _\vartheta }$, such that there exists an $\tau (x,y,\omega )\in \mathbb {N}$, with the following properties:

1. for all $$i \in \mathbb {N}, 0< i <\tau $$, $$(T_\omega ^i(x),T_\omega ^i(y)) \notin (\mathbb {T}^2\setminus \Delta _\delta )$$,
2. $$(T_\omega ^{\tau (x,y,\omega )}(x),T_\omega ^{\tau (x,y,\omega )}(y)) \in \Delta _{\kappa \delta }$$.

Here $\kappa$ as in the proof of Lemma 5.6.

For the lower bound in (5.10) we consider only orbit pieces near $\Delta$. From Lemma 2.5 we know that $(\mu _{\delta } \times \mathbb P) (\mathcal G) > 0$.

$$\begin{aligned} \mu ^{(2)}(\Delta _\varepsilon )&\ge \int _{\mathbb T^2 \setminus \Delta _\delta \times \Sigma _\vartheta } \sum _{i=0}^{\tau _{\delta ,+}(T_\omega (x),T_\omega (y),\omega )-1}1\!\!1_{(0,\varepsilon ]}(d(T^i_\omega (x),T^i_\omega (y))) \, d(\mu _\mathcal {K} \times \mathbb P) (x,y,\omega ) \\&\ge \int _\mathcal {G } \sum _{i=0}^{\tau _{\delta ,+}(T_\omega (x),T_\omega (y),\omega )-1}1\!\!1_{(0,\varepsilon ]}(d(T^i_\omega (x),T^i_\omega (y))) \, d(\mu _\mathcal {K} \times \mathbb P) (x,y,\omega ) \\&\ge \int _\mathcal {G } \sum _{i=\tau (x,y,\omega )}^{\tau _{\delta ,+}(T_\omega (x),T_\omega (y),\omega )-1}1\!\!1_{(0,\varepsilon ]}(d(T^i_\omega (x),T^i_\omega (y))) \, d(\mu _\mathcal {K} \times \mathbb P) (x,y,\omega ) \\&\ge \frac{\varepsilon ^{-\gamma }}{K(\kappa \delta )^{-\gamma }} \left( \mu _\mathcal {K} \times \mathbb P\right) (\mathcal G). \end{aligned}$$

We proceed with an upper bound. Here we must include orbits that start outside $\Delta _{R_\text {min}}$ and jump directly into $\Delta _\delta$, and also directly into $\Delta _\varepsilon$. This can be seen as pushing forward mass from $\mathcal {K}$ into $\Delta _\varepsilon$ in a single iterate. We divide the set $\mathcal {G}$ into two subsets, $\mathcal {G} = \mathcal {G}_d \cup \mathcal {G}_c$, with a set

$$\begin{aligned} \mathcal {G}_d = \mathcal {G} \cap \left( \mathbb {T}^2 \setminus \Delta _{R_\text {min}} \times \Sigma _\vartheta \right) \end{aligned}$$

and its complement $\mathcal {G}_c \subset \left( \Delta _{\delta /a_1}\setminus \Delta _\delta \right) \times \Sigma _\vartheta$.

Using this division, we get

$$\begin{aligned}  &   \mu ^{(2)}(\Delta _\varepsilon ) \le \int _{\mathbb T^2 \setminus \Delta _\delta \times \Sigma _\vartheta } \sum _{i = 0}^{\tau _{\delta ,+}(x,y,\omega )-1}1\!\!1_{(0,\varepsilon ]}(d(T_\omega ^i(x),T_\omega ^i(y))) \, d(\mu _\mathcal {K} \times \mathbb P) (x,y,\omega ) \\  &   \quad \le \int _{\mathcal {G}_c} \sum _{i = 0}^{\tau _{\delta ,+}(x,y,\omega )-1}1\!\!1_{(0,\varepsilon ]}(d(T_\omega ^i(x),T_\omega ^i(y))) \, d(\mu _\mathcal {K} \times \mathbb P) (x,y,\omega ) \\  &   \qquad + \int _{\mathcal {G}_d} \sum _{i = 0}^{\tau _{\delta ,+}(x,y,\omega )-1}1\!\!1_{(0,\varepsilon ]}(d(T_\omega ^i(x),T_\omega ^i(y))) \, d(\mu _\mathcal {K} \times \mathbb P) (x,y,\omega ). \end{aligned}$$

The first integral can be bounded from above by using Lemma 5.6. Recall that $f^+_{\varepsilon ,\delta }$ defined in (5.11) is a monotone function. Now

$$\begin{aligned}  &   \int _{\mathcal {G}_c} \sum _{i = 0}^{\tau _{\delta ,+}(x,y,\omega )-1} 1\!\!1_{(0,\varepsilon ]}(d(T^i_\omega (x),T^i_\omega (y))) \, d(\mu _\mathcal {K} \times \mathbb P) (x,y,\omega ) \\  &   \quad \le \int _{\mathcal {G}_c} f^+_{\varepsilon ,\delta }(d(T^{(2)}_\omega (x,y))) d(\mu _\mathcal {K} \times \mathbb P) (x,y,\omega ) \le f^+_{\varepsilon ,\delta }(\kappa \delta ) \le K \left( \frac{\varepsilon }{a_1 \kappa \delta }\right) ^{-\gamma }. \end{aligned}$$

To handle the second integral we consider separately the subset $\mathcal {G}_\varepsilon \subset \mathcal {G}_d$ of points that are mapped directly in $\Delta _\varepsilon$, thus

$$\begin{aligned} \mathcal {G}_\varepsilon = \left\{ (x,y,\omega ) \in \mathcal {G}_d \;; \; T^{(2)}_\omega (x,y) \in \Delta _\varepsilon \right\} . \end{aligned}$$

For $(x,y) \in \Delta _\varepsilon$ let, similar to (4.14.2),

$$\begin{aligned} \tau _{\varepsilon ,+}(x,y)&= \min \{n\in \mathbb N \; ; \; d(T_\omega ^n(x), T_\omega ^n(y))> \varepsilon \}, \\ \tau _{\delta ,+}(x,y)&= \min \{n\in \mathbb N \; ; \; d(T_\omega ^n(x), T_\omega ^n(y))> \delta \}. \end{aligned}$$

By Lemma 4.4 we have an estimate

$$\begin{aligned} \frac{1}{\lambda } \left( \ln \left( \frac{\varepsilon }{d(x,y)} \right) - 2K \right) \le \mathbb {E}\left[ \tau _{\varepsilon ,+}(x,y) \right] \le \frac{1}{\lambda } \left( \ln \left( \frac{\varepsilon }{d(x,y)}\right) + 2K \right) \end{aligned}$$

for some constant $K>0$. For $(x,y,\omega ) \in \mathcal {G}_\varepsilon$ we find $\tau _{\varepsilon ,+}(x,y,\omega ) < \tau _{\delta ,+}(x,y,\omega )$ almost surely. This allows us to write

$$\begin{aligned}  &   \int _{\mathcal {G}_d} \sum _{i = 0}^{\tau _{\delta ,+}(x,y,\omega )-1}1\!\!1_{(0,\varepsilon ]}(d(T_\omega ^i(x),T_\omega ^i(y))) \, d(\mu _\mathcal {K} \times \mathbb P) (x,y,\omega ) \\  &   \quad \le \int _{\mathcal {G}_\varepsilon }\left( \sum _{i = 0}^{\tau _{\varepsilon ,+}(x,y,\omega )-1} + \sum _{i = \tau _{\varepsilon ,+}(x,y,\omega ) }^{\tau _{\delta ,+}(x,y,\omega )-1}\right) 1\!\!1_{(0,\varepsilon ]}(d(T_\omega ^i(x),T_\omega ^i(y))) \, d(\mu _\mathcal {K} \times \mathbb P) (x,y,\omega ) \\  &   \qquad + \int _{\mathcal {G}_d \setminus \mathcal {G}_\varepsilon } \sum _{i = 0 }^{\tau _{\delta ,+}(x,y,\omega )-1} 1\!\!1_{(0,\varepsilon ]}(d(T_\omega ^i(x),T_\omega ^i(y))) \, d(\mu _\mathcal {K} \times \mathbb P) (x,y,\omega ) \\  &   \quad \le \int _{\mathcal {G}_\varepsilon } \tau _{\varepsilon , +}(T^{(2)}_\omega (x,y)) \, d(\mu _\mathcal {K} \times \mathbb P) (x,y,\omega ) + \int _{\mathcal {G}_\varepsilon } f^+_{\varepsilon ,\delta } (\varepsilon ) \, d(\mu _\mathcal {K} \times \mathbb P) (x,y,\omega ) \\  &   \qquad + \int _{\mathcal {G}_d\setminus \mathcal {G}_\varepsilon } f^+_{\varepsilon ,\delta }( d( T^{(2)}_\omega (x,y) ) ) \, d(\mu _\mathcal {K} \times \mathbb P) (x,y,\omega ). \end{aligned}$$

To bound the first integral in the final expression we use Lemmas 4.4, 2.3 and Hypothesis (H4) to get or any $0< s < 1$ the existence of a constant $\tilde{C} > 0$, such that

$$\begin{aligned} \int _{\mathcal {G}_d}\tau _{\delta , +}(T^{(2)}_\omega (x,y)) \, d(\mu _\mathcal {K} \times \mathbb P) (x,y,\omega ) \le \tilde{C} \varepsilon ^s. \end{aligned}$$

Hypothesis (H4) shows the existence of a constant $C>0$ with $(\mu _{\mathcal {K}}\times \mathbb P) (\mathcal {G}_\varepsilon ) < C \sqrt{\varepsilon }$. Lemma 4.4 shows that

$$\begin{aligned} f^+_{\varepsilon ,\delta } (\varepsilon ) \le C \frac{1}{\lambda } \end{aligned}$$

for some $C>0$. We get therefore a bound

$$\begin{aligned} \int _{\mathcal {G}_\varepsilon } f^+_{\varepsilon ,\delta } (\varepsilon ) \, d(\mu _\mathcal {K} \times \mathbb P) (x,y,\omega ) \le C \sqrt{\varepsilon } \end{aligned}$$

for some $C>0$. The third integral is bounded by

$$\begin{aligned}  &   \int _{\mathcal {G}_d\setminus \mathcal {G}_\varepsilon } f_{\varepsilon ,\delta }^+(d(T_\omega ^{(2)}(x,y)))\,d(\varsigma _\delta \times \mathbb P) (x,y,\omega ) \\  &   \quad \le \int _{\mathcal {G}_d\setminus \mathcal {G}_\varepsilon } K \left( \frac{\varepsilon }{d(T_\omega ^{(2)}(x,y))}\right) ^{-\gamma } \, d(\varsigma _\delta \times \mathbb P) \\  &   \quad \le K \varepsilon ^{-\gamma } \int _{\mathcal {G}_d\setminus \mathcal {G}_\varepsilon } \, d(T_\omega ^{(2)}(x,y))^{\gamma }d(\varsigma _\delta \times \mathbb P)(x,y,\omega ) \le C \varepsilon ^\gamma , \end{aligned}$$

for some $C>0$. Here we use Lemma 5.6 and Hypothesis (H4), and $\gamma < 1$. Combining the above analysis yields

$$\begin{aligned} \limsup _{\varepsilon \rightarrow 0 }\frac{\mu ^{(2)}(\Delta _\varepsilon )}{\varepsilon ^{-\gamma }}&\le C \end{aligned}$$

for some $C>0$, if $\gamma \in (-1/2,0)$. $\square$

## Footnotes

1 The moment Lyapunov function will be defined below, see Eq. (3.1) and Lemma 3.4.
